# Large-scale Models Reveal the Two-component Mechanics of Striated Muscle

**DOI:** 10.3390/ijms9122658

**Published:** 2008-12-18

**Authors:** Robert Jarosch

**Affiliations:** Formerly Institute of Plant Physiology, University of Salzburg, Hellbrunnerstrasse 34, A-5020 Salzburg, Austria. E-Mail: ilse.foissner@sbg.ac.at

**Keywords:** Muscle contraction, muscle mechanics, rotating filaments

## Abstract

This paper provides a comprehensive explanation of striated muscle mechanics and contraction on the basis of filament rotations. Helical proteins, particularly the coiled-coils of tropomyosin, myosin and α-actinin, shorten their H-bonds cooperatively and produce torque and filament rotations when the Coulombic net-charge repulsion of their highly charged side-chains is diminished by interaction with ions. The classical “two-component model” of active muscle differentiated a “contractile component” which stretches the “series elastic component” during force production. The contractile components are the helically shaped thin filaments of muscle that shorten the sarcomeres by clockwise drilling into the myosin cross-bridges with torque decrease (= force-deficit). Muscle stretch means drawing out the thin filament helices off the cross-bridges under passive counterclockwise rotation with torque increase (= stretch activation). Since each thin filament is anchored by four elastic α-actinin Z-filaments (provided with force-regulating sites for Ca^2+^ binding), the thin filament rotations change the torsional twist of the four Z-filaments as the “series elastic components”. Large scale models simulate the changes of structure and force in the Z-band by the different Z-filament twisting stages A, B, C, D, E, F and G. Stage D corresponds to the isometric state. The basic phenomena of muscle physiology, i. e. latency relaxation, Fenn-effect, the force-velocity relation, the length-tension relation, unexplained energy, shortening heat, the Huxley-Simmons phases, etc. are explained and interpreted with the help of the model experiments.

## 1. Summary of the most important results

**Figure 1:**
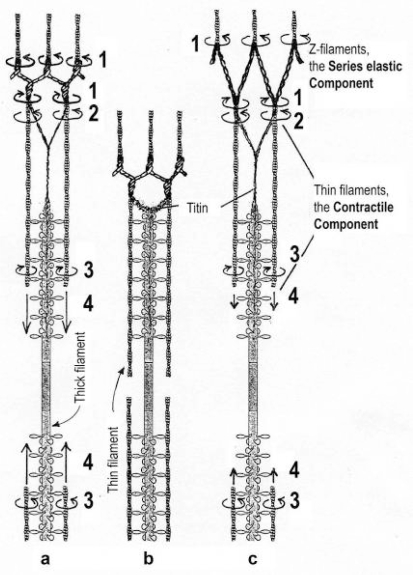
Diagram for the function of the two components in active striated muscle. The thin filaments are the “contractile component”. The Z-filaments are the “series elastic component”. Torque produces rotations of the Z-filaments (arrows 1) and thin filaments (arrows 2 and 3). The opposed torque of arrow l and arrow 2 is balanced. (a) to (b) Unloaded shortening. The distal parts of the thin filament helices rotate (arrows 3) and drill (= slide, arrows 4) into the myosin cross-bridges. No force arises, Z-filaments remain twisted, and the titin fibers shorten. (a) to (c) Isometric force arises when a heavy load prevents the distal filament drilling (arrows 4). The Z-filaments are elastically stretched and untwisted by the now predominant tensile stress and proximal torque (arrows 2) of the thin filaments. (c) to (b): A quick release of large amplitude results by thin filament drilling when the heavy load is reduced. The titin fibers shorten. The Z-filaments are drawn with exaggereted largeness. A scheme of J. Spudich provided for Stryer’s “Biochemistry” (third edition, Figure 36–19) was used as the basis for this diagram.

The classical “two component model” of active muscle assumed a “contractile component” that stretches the “series elastic component” during force production. According to the modern structural view the “contractile component” is represented by the thin filaments. Each thin filament is anchored by four Z-filaments which are the main “series elastic component”. In the diagram of Figure 1 the structural diversity is simplified by drawing two anchoring Z-filaments per thin filament only. Resting muscle shows the Z-filaments usually in the “small square” pattern, when seen in axial direction with the electron microscope. In the “small square” pattern the Z-filaments look swastica-like with bent cross-arms (see bottom of Figure 5). The bends have been formed probably during the last relaxation (see the end of this summary). After stimulation torque arises by Ca^2+^ activation in the α-helical coiled-coils (1) of α-actinin in the Z-filaments, (2) of tropomyosin in the thin filaments, and (3) in the myosin subfilaments. The torque results in torsional filament rotations. In the activated sarcomeres of Figure 1 filament rotations are indicated by arrows. At first after stimulation, the torque in the four Z-filaments twists them around (arrows 1), from the relaxed stage to stage A or B (compare Figure 5 and Figure 1a), and changes the Z-pattern from the “small square” to “basket weave” or “diagonal square”. The torque of the two tropomyosin coiled-coils twist the actin filaments, as indicated by arrows 2 and 3 in Figure 9e. The free distal ends of the thin filaments can now rotate in clockwise direction as seen from the Z-band (arrows 3 in Figure 1a). The counterclockwise rotation of the anchored proximal ends (arrows 2) is equilibrated by the torque (arrows 1) of the four Z-filaments.

**Figure 2:**
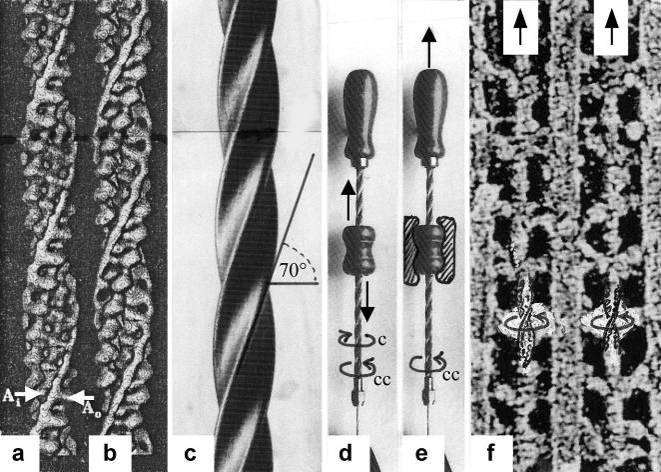
The thin filaments slide by using the mechanics of a drill-borer. (a) Surface view of a reconstruction of a thin filament of frog skeletal muscle in EGTA, and (b) of *Limulus* muscle in Ca^2+^. The bright structures wound about the right-handed helical actin filaments are the two tropomyosin coiled-coils, that in (b) are shifted a little to the left. Arrow A_0_ indicates the position without Ca^2+^, arrow A_i_ the position with Ca^2+^ (from Vibert *et al*. [35], with permission). (c) Screw of a right-handed drill-borer, shown in (d) and (e). The inclination angle of the thin filament helix and drill-borer screw is about 70°. (d) The drill-borer works by moving up and down (arrows) the handle with a nut-coil. This causes clockwise and counter-clockwise rotation (arrows) of the screw. (e) When the handle with the nut-coil is fixed, pulling up the screw (arrow up) causes counter-clockwise drilling rotation (arrow cc), as seen from above. (f) High EM magnification of two thin filaments on both sides of a thick filament (“myac” view, Z-band is topwards) of an insect flight muscle in rigor (quick-freeze and deep-etched preparation of John Heuser [36], with permission), When ATP is added to an intact rigor muscle, a stretch can pull out the thin filaments off the cross-bridges (arrows up). That means a counter-clockwise drilling rotation of the thin filaments (arrows cc) as demonstrated by the drill-borer model in (e).

**Figure 3:**
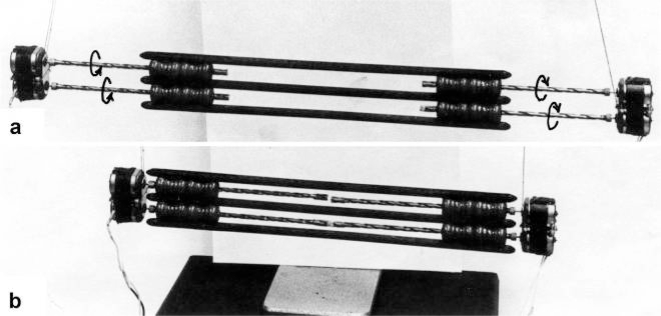
Electromotor-driven model for the sarcomere contraction. It consists of four combined drill-borer. Each borer screw rotates about 2.5 times (arrows). From Jarosch [22].

**Figure 4:**
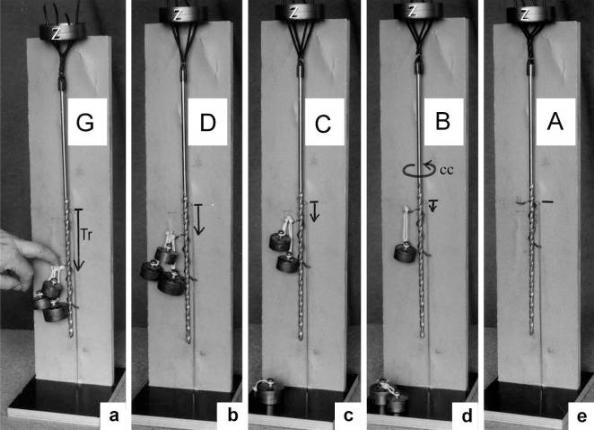
The adjustment of the Z-filament twisting stage by the load. This model demonstrates that increased load enhances the amount of the helix rotation (arrow cc in (d)) and changes the clockwise Z-filament twisting stage from A (zero g), B (100g), C (200g) to the untwisted Z-filament stage D (400g), and anticlockwise twisting in G, produced by strong finger pressure. Tr =translocation of a “cross-bridge” against the filament.

**Figure 5:**
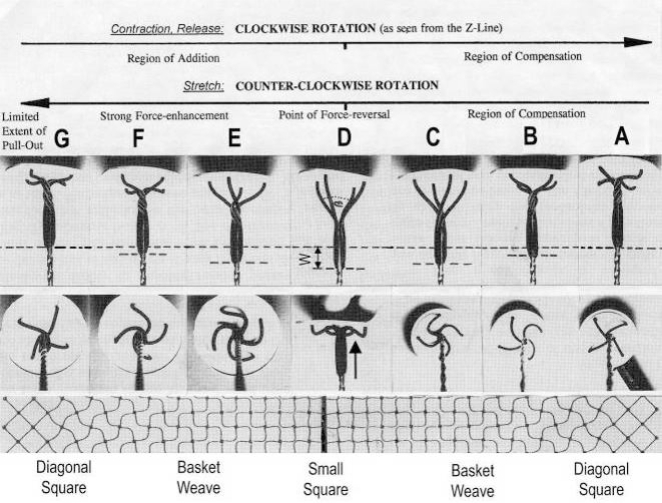
All twisting stages of the Z-filaments from A to G are shown in side view (upper panel) and axial view (middle panel). Only stage D is here shown in side view also in order to demonstrate that the swastica look with bent cross-arms results by shifting the helix to the Z-disk (arrow in stage D). “Region of Addition” and “Region of Compensation” indicate where the winding distance W (shown in D) increases or decreases the amount of sliding. The Z-filament patterns “small square”, “basket weave” and “diagonal square” appear in the electron microscope when seen in axial direction. These patterns are drawn in the lowest panel according to Yamaguchi *et al*. ([53], with permission). For more details see text. Improved from Jarosch [50].

The Z-line directed tips of the myosin subfilaments drill out of the thick filaments and contact the thin filaments as the cross-bridges. What now follows is substantially determined by the amount of muscle load. The activation of an unloaded sarcomere leads from Figure 1a to Figure 1b. The rotation (arrows 3) of the right-handed thin filament double helices (reconstructed electron micrographs are shown in Figure 2a, b) takes place as a drilling (=sliding) motion into the myosin cross-bridges (arrows 4 in Figure 1). The sliding process may be supported by winding up and unwinding of myosin S_1_ α-helices. ATP can be split during unwinding when it is bound by myosin and F-actin. A simple electromotor-driven model simulates this sarcomere shortening (Figure 3). The isotonic shortening velocity of the drilling motion in muscle is determined by the amount of load that is lifted according to the force-velocity relation (Figure 11d). The indicated Z-filament twisting stages (A, B, C and D) depend on the load as shown by the model of Figure 4, which is burden by increasing weights. This model demonstrates that the twisting stage of the series elastic Z-filaments is automatically adjusted by the load. Together the amount of bound Ca^2+^ at the free non-twisted Z-filaments increases from stage A to the isometric stage D, as indicated by the relaxed and stretched model of Figure 10. This means a load-dependent increase of torque, force, and muscle work between stage A and D that explains the Fenn-effect. However the twist of the Z-filaments increases further from stage D to G, but the free binding sites for Ca^2+^ decrease again from D to G, as shown by Figure 5.

**Figure 6:**
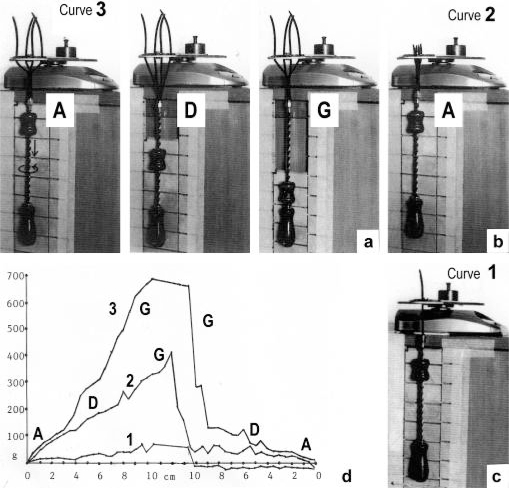
Drill-borer models demonstrate the force produced by the Z-filaments during unwinding and winding up from stage A to G and back to A. The disk with the Z-filaments and drill-borer was fastened on a digital balance and the force (g) produced by stepwise shifting down the drill-borer handle was measured. The curves in (d) show that the strongest pull is produced when a larger distance separates the single Z-filaments. The square distance was 30 mm in (a) (curve 3 in (d)), and 7 mm in (b) (curve 2). Twisting a single Z-filament, shown in (c), produced weak pull only (curve 1).

**Figure 7:**
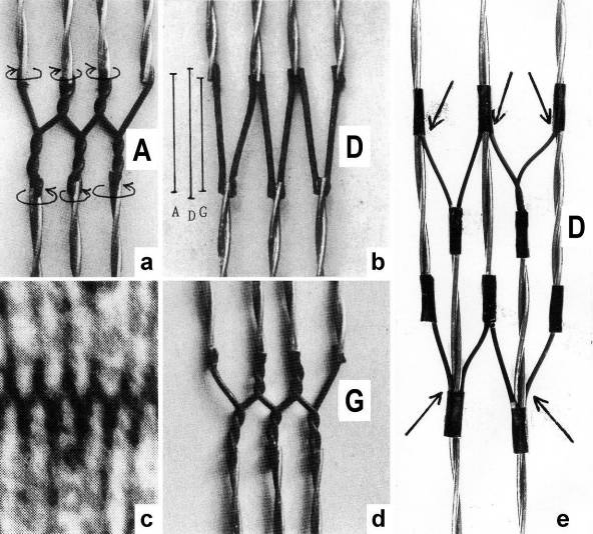
Z-band dynamics. Two Z-filaments only are used per thin filament. (a), (b) and (d) Models of narrow Z-bands with one zigzag layer only. The relaxed stage A is also shown in (c) from a guppy muscle (electron micrograph from Yamaguchi *et al*. [53]; with permission). Note that the structure of the isometric stage D in (b) has a weaker consistence against lateral influences than the stages A or G. (e) Model with two zigzag layers (from Jarosch [74]). The arrows indicate the sites where Z-filaments can wind about thin filaments. For more details see the text.

Muscle tension arises only when shortening is hindered by a load. Isometric force arises from Figure 1a to Figure 1c by thin filament rotations (arrows 2) but only when shortening (arrow 4) is fully prevented by clamping the muscle ends or by a heavy load. At the start of activation the thin filament helices cannot drill but should rotate (arrow 3) at first quickly on the same place (“idle rotation”), since the torque is not yet balanced by the resisting cross-bridges. Here, the helical turns run along to the Z-bands as helical waves. This means that the initial cross-bridges slip against these helical waves and produce “unexplained heat” by friction. With increasing number of contacting cross-bridges, torque and tensile stress arises strongly in the thin filaments and put up the balance. The effect of stretch on the Z-filaments was already shown by the model of Figure 10. Together they are turned by the increasing thin filament torque from stage A to D into the direction of arrows 2 in Figure 1a. This occurs against the increasing Z-filament torque (arrows 1) and leads to an isometric balance between the torques. Thus the isometric force is the result of a heavy load that produces strong torque and tensile stress which is stored by untwisting (arrows 2) the Z-filaments. The increasing force is demonstrated by the model of Figure 4 and by the drill-borer models of Figure 6a, b. The resulting Z-band dynamics is shown by the models of Figure 7, where also two Z-filaments per thin filaments only, were used.

Increased tension results also when an activated muscle is stretched (“Force enhancement by Stretch”, “Stretch activation”), because the thin filament helices are drawn out off the cross-bridges under counterclockwise drilling rotation (arrow cc), as demonstrated by the drill-borer model of Figure 2e. Torque and tensile stress of stretch untwist the Z-filaments from stage A to D and further to stage E, F or G as shown by the models of Figs. 4 and 5.

Stretch can produce strong muscle tension, also without activation, presumably because some resisting cross-bridges may be present in resting muscle.

When a prestretched muscle with long sarcomeres is activated and quickly released from stage G, F or E, the distance W, shown in Figure 5D and Figure 10a, or a fraction of it, is at first released by unwinding the Z-filaments and causes “latency relaxation”, before shortening from stage D to A (structural change from Figure 1c to Figure 1b) takes place.

In order to study the mechanics of length changes more in detail, another model (Figure 14) was used. It contains the “contractile” and “series elastic component” combined as a unity. The “contractile component” is represented by a double helix containing of two intertwined rubber cords. The model is fully relaxed in Figure 14a, twisted by the torque for 16 rotations in Figure 14b, and twisted and loaded by 400 g in Figure 14c. The torque for the 16 rotations of the free end is equilibrated by the torque for about 1 rotation of the Z-filaments.

The tension-curves of some quick releases of a frog muscle in the isometric stage are shown in Figure 15a. The release amplitude increases from very small (0.6 mm) to very large (11.7 mm). Small quick releases with amplitudes of 1.5 nm to 6 nm per half sarcomere are recorded in Figure 16a. The structural change of a large quick release is depicted from Figure 1c to Figure 1b. At first the Hookenian elasticity of the tensile stress is quickly relaxed (Huxley-Simmons phase 1, Figure 22). The tension falls to zero after about 6 nm release distance per half sarcomere (Figure 16a). The initial quick relaxation is connected with a twist change of the Z-filaments, e.g. from stage D in Figure 18a to <C in Figure 18b, when the release amplitude is small. With larger amplitude the level of the Hookenian elasticity is passed over, and the thin filaments become at first slack, as demonstrated by Figure 19b. But the strong torque in the thin filament is not affected. It starts at first clockwise rotation (arrow 3) and drills (arrow 4) a limited distance that depends on the release amplitude. When this drilling is stopped the proximal torque (arrow 2) can unwind the Z-filaments and produces again tensile stress which appears as the redeveloped force (Huxley-Simmons phase 2 in Figure 22). Some Ca^2+^ bound by the free Z-filament sites in Figure 18a and Figure 19a is displaced as “extra Ca^2+^” from the Z-filament region twisted around (straight arrows in Figure 18b, Figure 19b, and after a second release in Figure 19c). As a consequence the Z-filament torque (arrow 1) is transiently reduced or reversed (Figure 18b and Figure 19c), so that a delayed force decrease is observed (Huxley-Simmons phase 3, Figure 22). When this occurs the direction of the proximal torque (arrow 2) of the thin filaments can easily untwist the Z-filaments during force recovery, e.g. from stage <C in Figure 18b to >D in Figure 18c. Here, a very small amount of torque is lost. A larger quick release, e.g. 6 nm per half sarcomere (tension curve 6 in Figure 17) displaces more Ca^2+^ (as indicated by the higher number of straight arrows) than a smaller one, e.g. l.5 nm per half sarcomere (tension curve 1.5 in Figure 17). Therefore recovery is more rapid from large releases with steeper slope of the tension curves, than from small ones (Figure 17). A feedback controlled damped twist oscillation of the Z-filaments (indicated in Figure 18b and Figure 19c) can also occur by displacement and rebinding of Ca^2+^. These oscillations are typical after a quick release of length or load (Figure 15a and Figure 23a, b). “Extra Ca^2+^” displaced from the Z-filaments after a quick release of high amplitude (e.g. Figure 22d) can be bound by troponin C and may again strengthen the thin filament torque and force (Huxley-Simmons phase 4). This “positive feedback”, e.g. after five quick releases in Figure 20d, delays “force deficit”, and may be important for “prolonging the active state” during relaxation.

After a quick stretch the Huxley-Simmons phases show the opposed direction of release (Figure 22b, c). In the high-tension region between Z-filament twisting stage D and G, length changes lead to irregular behaviour of the force-curves (Figure 24) that may depend on the left-handed winding direction of the Z-filaments (Figure 25).

When the load is quickly reduced from isometric conditions to constant levels (Figure 23) stretch relaxation and twisting change of the Z-filaments occur at first as described for the quick release. But then a clockwise drilling rotation of the thin filaments (arrows 4 in Figure 1) shortens the sarcomeres with steady state velocity that increases with decreasing load, as shown by the increasing slopes of Figure 23. This motion is analogous to the lifting of a load by a rotating screw with constant torsional force (Figure 11a, b). But the initial quick load reduction in Figure 23 has changed also the Z-filament twisting stage from D to C or B, as indicated. This means decreased force and torque from stage D to A. Therefore the force-velocity curves of muscle are hyperbolae (Figure 11c, d, e) and not straight lines as the lifting curves of a load with constant torsional force in Figure 11b, because the torque increases with the load from stage A to D, as indicated in Figure 11d.

The thin filaments contain a great reservoir of torque. But torque is lost by each active thin filament rotation (arrows 3 and 2 in Figure 1). This results in force “depression” or “force-deficit”. The redeveloped force after a large release does not more reach the original level. Extreme “force depression” is obtained by the extensive release in a “slack test” (see the lower tension curves of Figure 15a and the curves of skinned fibers in Figure 15b).

Relaxation starts when the stimulus ceases. Ca^2+^ is withdrawn from the filaments to the sarcoplasmic reticulum. The torque in the coiled-coils disappears, but the remaining torsional tension in the twisted actin filaments can drill them back in counterclockwise direction to the Z-bands. This means some restretching of the sarcomeres and pushing (arrow in Figure 5D) the untwisted Z-filaments of the relaxed stage D to the “small square” pattern by bending the cross-arms (see the beginning of that summary).

## 2. Introduction: The basic molecular event for filament sliding and force generation

According to the conventional theory the subfragment S1 of the enzyme myosin supplies the force for actin filament sliding during muscle contraction by an ATP-dependent “working stroke” of the “lever arm” (see for example Holmes in Sugi’s book, p. 13 [1]). The idea of cycling cross-bridges persisted since 40 years. However the molecular details of the “working stroke”, its velocity, its force, the viscous resistance of the medium, etc., are obscure. Prof. Sugi writes in the preface of this book “...we cannot give a clear answer to the question “what makes the filaments slide?”” [2]. A clear answer and plausible explanation of sliding results by the assumption that the thin filaments are rotating helices which can drill into the myosin cross-bridges, as a cork-screw drills into the cork (see chapter 3.1). However, one must not overlook the two extreme kinematical possibilities of a rotating helix: (1) Rotation on the same place (“idle rotation”) with helical waves into the opposite direction of drilling, when the medium is without sufficient resistance. (2) Drilling (= sliding), when a dense medium or many cross-bridges strongly resist or prevent the helical waves. What kind of rotation takes place should depend on the number of adjacent cross-bridges and on muscle load. Of course, there are also many states of transition with both kinds of motion: drilling and waves. For example in vitro sliding of actin filaments over a myosin lawn in the presence of ATP should depend on an effective drilling motion. When they undergo the characteristic “wiggling” motions without displacement [3] slipping against the substratum may take place under idle rotation.

A reviewer suggested that I should explain why I did not perform biological experiments on muscle: I started my investigations in plant physiology with my doctor work on cytoplasmic streaming [4]. At the same time (1954) when the classic papers on filament sliding in muscle were published [5,6] I discovered motile fibrils in living cytoplasmic droplets squeezed out of characean internodal cells and observed them with optimal darkfield [4, 7–11]. Very fine fibrils visible by adhering particles moved quickly by repelling the immediate vicinity backwards into the opposite direction. The fibrils formed quickly rotating loops which wound up into ring bundles. The ring bundles inclined to become polygons with 4, 5, 6 or more angles. The crucial phenomenon was a regular wave-like motion of the angles and of the whole polygonal shape along the fibrillar ring bundle with its installed motionless particles. This wave-like motion was at first a mystery. But it could be explained, since the double-helical structure of actin filaments was revealed by electron microscopy [12], and because the idle rotation of a helix shows the helical turns always in a wave-like motion. When actin filaments are mutually wound up and unwound a wave-like motion of the angles must result that could be simulated by helical spring models [13, 15, 17].

The author concluded that active cytoplasmic streaming is the displacement along bundles of quickly rotating actin filaments [13, 14, 16]. *In vitro* water streams along parallel actin filaments could also be produced when myosin and ATP was added [18, 19, 20]. Oplatka [21] suggested rotors at the heart of all biological motors. But this interpretation was not accepted because in the meantime the idea of cycling cross-bridges, developed on muscle, was applied to other kinds of cell motility. So I turned to muscle in order to work out a better interpretation for its function than the swinging cross-bridge theory, which cannot give plausible explanations for many important facts of muscle physiology, e.g. stretch activation, force-depression after release, latency-relaxation, Fenn-effect, auto-oscillatoric behaviour, etc.. The concept of rotating filaments yields a plausible basis for all these phenomena.

Why do the thin filaments rotate? Charged α-helices and coiled-coils produce torque and torsional filament rotations by cooperative shortening of their H-bonds (see Chapter 3.6). Stretch of the compliant H-bonds is the result e.g. of the Coulombic repulsion between negatively charged side-chains arranged in axial direction. When cations are bound, the negative charge is diminished and H-bonds shorten cooperatively [22–24]. Ca^2+^ was shown by Bacchiocchi and Lehrer [25] to produce torsional tropomyosin rotations that should twist the actin filaments. Thus torque transmission seems to be a property of charged α-helices. The myosin subfragment S1 only can support actin filament sliding motion [26, 27], presumably by winding and unwinding of α-helical chains. The work of Nishizaka *et al*. [28] and Sase *et al*. [29] demonstrated that torque for *in vitro* rotation and twisting of actin filaments is induced by myosin cross-bridges. Addition of the troponin-tropomyosin complex increases the sliding velocity and force [30–34] presumably by winding and unwinding of the tropomyosin coiled-coils. This mechanism may also answer the questions, why the coiled-coil α-helices are wound about each other and why the two tropomyosins are wound about the actin filament. Thus a basic rotational mechanism seems to work and may replace the conventional conception of the “power” or “working stroke”.

It will be shown by future work whether the torque production in α-helical proteins is sufficient to explain the filament rotation of the various kinds of biological motions or whether it is necessary to assume other physical forces, e.g. such as proposed by Tirosh (this volume).

## 3. Results

In the following chapters the rotations of muscle filaments and their effects are described with the help of mechanical large scale models. These motions are partly very complex, so that a poor imagination without models would be incomprehensible.

### 3.1. Stretch activation: Torque-increase by passive rotation of the thin filaments. Isotonic contraction: Torque-decrease by active drilling (= sliding) into the myosin cross-bridges

The conventional theories of cycling cross-bridges neglect the helical shape of the thin filaments. Figures 2a and b shows the right-handed double helical structure of thin filaments from frog and *Limulus* muscle, reconstructed from electron micrographs by Vibert *et al*. [35]. When these helices contact many cross-bridges (with or without position changes of the lever arms), they must rotate and drill by the same mechanical reasons why a cork-screw must rotate and drill in the cork-stopper. It cannot move without drilling, except by cork-destroying violence. This mechanics is still better demonstrated by a drill-borer. The drill-borer screw (Figure 2c) has about the same inclination angle (near 70°) as the thin filaments of muscle. A drill-borer works by moving up and down a handle (straight arrows in Figure 2d), which operates like a nut-coil and produces a periodical change of the screw’s rotational direction (arrows c and cc). Thus the drill-borer transforms axially directed motion to rotation. 70° seems to be the best (optimized) inclination for this purpose. There are drill-borers either with left- or right-handed screw sense. If the handle of a right-handed one is fixed (Figure 2e), pulling upwards the screw (arrow up) produces counterclockwise drilling upwards (arrow cc, as seen from above). Moving down the screw means clockwise drilling down. The myosin cross-bridges of muscle contact the thin filaments as shown e.g. by an insect flight muscle in rigor (Figure 2f, electron micrograph of a quickly frozen preparation of Heuser [36]). When such a living muscle is activated and forcibly stretched in the presence of ATP, the thin filaments are pulled out off the cross-bridges in the direction of the straight arrows in Figure 2f. This must result in a similar counterclockwise drilling rotation of the thin filaments as performed (arrow cc) by the borer screw of Figure 2e. Since each thin filament is anchored by four Z-filaments, this counterclockwise rotation increases torque and tension not only of the thin filaments, but also of the Z-filaments and explains “Stretch activation” [37]. The significance of the Z-filaments was largely underestimated by the cross-bridge theories, because the origin of force was assumed in the cross-bridges. Indeed the Z-filaments are the most important “series elastic component” of muscle that was postulated by the “two-component model” of Levin and Wyman [38] and Hill [39,40]. Activation and shortening of the “contractile component” stretches the “series elastic component” ([39], compare the model of Figure 10).

As described, the helical thin filaments (the “contractile component”) transform the axially directed motion of stretch to rotation. The strong torque of stretch stored in the Z-filaments is so used during release for clockwise drilling rotation of sarcomere shortening. Also without stretch, after stimulation by Ca-activation, the helical thin filaments transform their tropomyosin generated torque into the axially directed drilling rotation of shortening. Myosin S_1_ chains may strengthen the drilling motion by winding up and unwinding with ATP-splitting.

The large scale rotational motions of the thin filaments in muscle have been monitored, e.g. using steady-state phosphorescence emission anisotropy that increased upon binding of S1 in the presence of ATP (see [41] and further literature therein). “Long axis torsional motion is the only possible large scale rotational motion with an entangled filament network such as F-actin” [42]. As already mentioned in the introduction recent FRET-data indicate that Ca^2+^ causes tropomyosin to rotate about its axis [25]. Since two tropomyosin coiled-coils are bound and wound about each actin filament, the tropomyosin torque twists the actin filament. Therefore “tropomyosin promotes a conformational change in actin that is energetically unfavourable in the absence of tropomyosin” [43, p. 27592). The tropomyosin motion increases the intensity of the second actin layer line in X-ray diagrams (see chapter 3.6), but the F-actin structure remains at first unchanged when torque is produced and stored. A twist- and pitch-change can only occur later when it is realized by the torsional rotations of the thin filaments (compare e.g. Figure 19b to c).

The model of Figure 3, from [22], shows the function of the “contractile component”. It simulates sarcomere shortening by about three electromotor-driven clockwise drilling rotations (arrows) of drill-borer helices. But this model cannot explain muscle force because it lacks the series elastic Z-filaments that accumulate torque and tensile stress (see the next chapters).

The high inclination angle 70° of the F-actin helix may be important for muscle energetics. It means that during muscle stretch nearly the full kinetic stretch energy is transformed into counterclockwise filament rotation. This is in contrast to sarcomere shortening, where some of the kinetic energy of clockwise rotation is dissipated by frictional heat. Shortening heat is not due to ATP splitting [44,45]. It is described as energy resulting from an “unidentified process” ([46–48], quoted from [49]). Obviously this process is the clockwise drilling motion of the thin filaments during isotonic shortening. The frictional resistance and its heat production would be similar during stretch and shortening, if the helical inclination angle of F-actin would be 45°. The actual inclination of 70° strongly favours stretch activation, i.e. force enhancement, heat production during shortening (see Chapter 3.11), and slack-removal of passive muscle (see Chapter 3.5).

### 3.2. The twisting stages A to G of the four anchoring series elastic Z-filaments

Since each thin filament is anchored by four Z-filaments, the counter-clockwise rotation of the thin filaments during stretch (arrows cc in Figures 2e, f) produces or increases torque by twisting or untwisting the four Z-filaments. The increased torsional stiffness of the activated thin filaments (see Chapter 3.6) should prevent passive stretch-induced twist of the thin filaments. To study the intricate elastic behaviour of the Z-filaments a functional model was used. It consists of a right-handed borer that represents the thin filament, a plastic disc, and four pieces of rubber cord as the elastic Z-filaments. The rubber cords are fixed by super glue at the non-helical end of the borer. The other ends of the rubber cords are then put into four suitable holes in the plastic disc so that they cannot slip in the holes. Now the single rubber cords are turned in the holes in clockwise direction (as seen from above) by about one full rotation. The result is the right-handed twisting stage A (Figures 4e and 5A). The model is fixed in hanging position. A helically bent soft wire wound about the borer helix works like a nut-coil and rotates the borer in counter-clockwise direction (arrow cc in Figure 4d) when it is shifted down (straight arrows) by weights or finger pressure. A straight steel pole guides this motion. The borer rotation changes the twisting stage of the four Z-filaments. All possible twisting stages are shown in Figure 5 [improved from 50]. Counter-clockwise rotation during muscle stretch moves the Z-stages along the long arrow to the left. The right-handed twisting stage A is unwound over B and C to the untwisted stage D. Further stretch leads to the left-handed twisting stages E, F and G. Stage G is the “limited extent of pull out” [51, 52]. Clockwise rotation means shortening or release of muscle (long arrow to the right), and can lead from stage G over D to A. The different twisting patterns of the Z-filaments seen in the electron microscope when observed in axial direction were named “small square”, “basket weave” and “diagonal square”. They are shown on bottom of Figure 5 according to Yamaguchi *et al*. [53]. Note that the Z-filament cross-arms between the tetragonally arranged axial filaments are longest in the “small square”, become shorter in the “basket weave” and shortest in the “diagonal square”. The upper panel of Figure 5 explains this behaviour because a part of each cross arm is wound up. The “small square” with its bent Z-filaments is frequently found in resting muscle [54–58]. Ca^2+^ binding starts 1–2 ms after the onset of a stimulus, and most troponin molecules have bound Ca^2+^ at 4 ms [59, 60]. Since Ca^2+^ comes out from the T-tubules in direct vicinity of the Z-band, it is likely to be bound to the Z-filaments within the first millisecond. The produced torque should twist the Z-filaments from the tension-less resting stage (“small square”) to stage A. This motion may take place without peculiar resistance since the thin and thick filaments are not yet activated. However, the swastica look of the four Z-filaments in the “small square” and “basket weave” appears on the model only when the screw is shifted into the direction of the arrow shown in the middle panel of stage D in Figure 5. This shifting into the direction of the Z-band may take place during muscle relaxation when most Ca^2+^ is withdrawn and the Z-filaments untwist to the relaxed stage D_r_. The tropomyosin torque disappears and the remaining tension in the twisted actin filament helices can drill them in counter clockwise direction backwards to the Z-band. Ramsey and Street [61] described first that isolated muscle fibres, lying in Ringer’s solution, reextend themselves to the original slack length after a twitch or short tetanus. They proposed that the relaxation was an active process. Hill [62] questioned the activity of this reextension, but it was again described by other scientists [63, 64]. Gonzalez-Serratos [65] brought straight fibres into a block of gelatine (length about 2.6 μm). When the sarcomere length was reduced to about 2.0 μm by compressing the gelatine block, the fibre as a whole remained straight but the myofibrils became wavy which confirmed the reversed sliding movement of the filaments [see also 66].

### 3.3. Isometric muscle force arises by stretch and unwinding the four Z-filaments from stage A to D

“The isometric response of muscle as ordinarily recorded, depends on the shortening of its contractile elements against the rising tension of its elastic elements, and that is a slow process. An applied stretch obviates the necessity of shortening and allows the change of stage resulting from stimulation to be examined directly” [67, p. 320; 68, p. 70].

It is shown by the model of Figure 4 that the increasing load from stage A (zero), B (100g), C (200g) and D (400g) stretches and untwists the Z-filaments. The stretch is also effective when the thin filaments (the “contractile component”) drill into the myosin cross-bridges by lifting a load during isotonic shortening. Here the load determines the twisting stage (e.g. B or C) and the shortening velocity, according to the force-velocity relation (see chapter 3.9). Under isometric conditions drilling of the thin filaments is limited and stretches the Z-filaments slowly to about stage D.

To comprehend more exactly the amount of the produced tensile stress under different stretch conditions a drill-borer with the rubber Z-filaments was fixed on a digital balance (Figure 6). The drill-borer handle with the nut-coil was shifted down 12 cm in steps of 0.5 cm, guided by a wooden rail. The amount of pull (g) after each step was read off from the balance display. As shown by curve 1 in Figure 6d a single rubber Z-filament (Figure 6c) produces little pull (max. 80 g) when it is twisted by the drill-borer. Four Z-filaments in a distance of 7 mm (Figure 6b) produce much more pull (max. 400 g in stage G) during untwisting and twisting the Z-filaments (curve 2 in Figure 6d). The same Z-filaments in a distance of 30 mm (Figure 6a) produced most pull (about 700g in stage G) as shown by curve 3 in Figure 6d. The curves are not smooth because rubber has long-lasting after effects. The strong fall of the curves 3 and 2 after the peaks from stage G to D may depend on lengthening (unwinding) the distance W shown on the models of Figs. 5D and 10a. As demonstrated by Figure 6a and b an important factor for the production of strong tensile stress during untwisting and twisting is a large distance between the Z-filaments. We found in chapter 3.1 that the helical structure of the thin filaments transforms the tensile stress of stretch into counter clockwise rotation of the thin filaments. This rotation is now back-transformed in the Z-band and supports and accumulates tensile stress by an ingenious trick: The counter clockwise thin filament rotation is used for unwinding the four distant Z-filaments in order to generate the strong isometric force.

### 3.4. Z-band dynamics and the “latency-relaxation”

Each of the four Z-filaments is connected with another thin filament of the neighbour sarcomere as was shown by Knappeis and Carlsen [69], Franzini-Armstrong and Porter [70] and Reedy [71]. These connections hold the thin filaments near the Z-band in a tetragonal order. The Z-band is so like a fusion of many models as shown in Figure 4, but with a superimposed dynamics: The motions on both sides of the Z-line are related like image and mirror-image. This was already observed by A. Huxley [72, p. 278], when he performed local stimulation experiments on frog muscle by applying an electrical pulse to a micropipette in contact with a fibre near the Z-line: “We never saw a contraction involving one half of an I-band only”. The experiment suggests “that the functional units consist of a Z-line with the I-band in which it lies and the half A-band on either side”. This observation appears as the result of a balanced Z-filament torque that works alike on both sides of the Z-line. Huxley’s later experiments “showed that the sensitive area in crab mucle was close to the A-I boundary; depolarization at this point caused shortening of the adjacent half I band” [quot. 105, p. 330]. This connection of the sarcomeres by the highly elastic Z-filaments that transmit the torque must be the basis of the synchronization phenomena described by Ishiwata *et al*. [73].

It is difficult to demonstrate the Z-band dynamics by a model with four Z-filaments per thin filament. Therefore Figure 7 shows simplified Z-band models in side view with two Z-filaments per thin filament only. Figures 7a, b, d present models of a “narrow Z-band” with one zigzag layer as found in fish body white muscles. Figure 7e [from 74] shows a model with two zigzag layers and overlapping actin filaments. Here and when more zigzag layers exist, Z-filaments can wind about the actin filaments at sites indicated by arrows. The “wide Z-bands” consist of three or four zigzag layers, e.g. in cardiac muscles and in slow muscles as found in bovine neck muscle and rat soleus [75]. Figure 7a presents a model of the tensionless A-stage that is shown in reality in Figure 7c by a guppy muscle (electron micrograph of Yamaguchi *et al*. [53]). Untwisting the A-stage (arrows) by about one counterclockwise rotation of the proximal thin filament ends leads to the untwisted stage D in Figure 7b. A strong stretch produces further counter-clockwise rotation, leading to stage G, shown in Figure 7d. The change from A to D means slow rise of the isometric force by overcoming the torsional resistance of the Z-filaments. As indicated by the bars A, D, G in Figure 7b stage D exhibits longer Z-filaments and little thicker Z-bands than stage A and G. The length difference is the double distance W shown at the models of Figs. 5 and 10. During Ca^2+^ induced sarcomere shortening under isometric conditions the thin filament rotate at first in clockwise direction and drill into the myosin cross-bridges. When shortening is not more possible, the drilling motion (arrow 4) is stopped, and the rising tension and rotation (arrow 2) has stretched the Z-filaments to the isometric stage D. The model of Figure 10 demonstrates that such a stretch can bring about this effect. Also other series elastic elements of muscle (thin, thick filaments and tendons) may be stretched, but there is no doubt that the main series elastic components distributed along the muscle fibres ([220, p. 538]) are the Z-filaments.

A reviewer states that the unwinding of the Z-filaments in Figure 1 seems to widen the Z-line structure, implying an elongation of the sarcomere length which contradicts to the condition of isometric contraction where the sarcomere length should be maintained constant. Figure 1 is a diagram drawn with largely increased Z-filaments because of better appreciation. The real elongation of a vertebrate muscle sarcomere under isometric tension is 2 times the distance W in Figure 5, but may be not more than a few nanometer. Moreover, the isometric tension stretches the thin and thick filaments too. Thus a length measurement of a muscle sarcomere fixed in the isometric state will hardly yield the elongation of the Z-filaments. But Akimoto and Sugi [76] have shown on the very large sarcomeres (length about 7μm) of living horseshoe crab skeletal fibres, that the A-band length during Ca^2+^ induced shortening increases by thick filament misalignment. Together they found a tendency of the Z-bands to become thinner. Obviously, this means that the Z-filaments shorten from the isometric stage D to stage A by winding up.

The change from stage A to D during rise of isometric tension, or muscle stretch, should be connected with softening of the muscle structure. It is shown by stage A and G of Figure 7 that the thin filaments are mechanically much stronger connected by the zigzag Z-filaments than in stage D. Besides, increased spacing between the thin filaments was found in the isometric stage [77,78] that is not shown by the models of Figure 7. Hanson and H. Huxley [79, 80] already suggested the localisation of the “series elastic component” near the Z-line, since after stretch experiments with low ATP concentrations the Z-line became less dense. Tamura *et al*. [81] measured stiffness changes by recording the propagation velocity of ultrasonic waves (3–7 MHz). With this method Hatta *et al*. [82] described the whole stiffness curve during a tetanus. Here, the transverse stiffness, recorded by waves in perpendicular direction to the fibre axis, decreases during the isometric plateau, indicating softening of the muscle structure as also noted on the model of Figure 7b.

Changes of the optical transparency as found by D. K. Hill [83] during a twitch in stretched frog’s skeletal muscle may also interpreted by the described structural changes. For example, Hill’s early rapid phase of increased transparency should depend on the transition from stage G in Figure 7d, over stage D in Figure 7b (with the more distant filaments), to stage A in Figure 7a.

The transition from stage G to D is also the basis of a very small transitory lengthening preceding the usual shortening of muscle, discovered by Rauh [84] and described as a “nose” in his myograms. It was also found by Fischer [85], reviewed by Schaefer and Göpfert [86], and named “latency-relaxation” by Sandow [87]. A. V. Hill found no such elongation in muscles under very low tension and concluded [67, 88] that it must arise in a structure parallel to the contractile system. He suggested a similar process for the early increase of transparency and latency relaxation [67]. A. Huxley [72] assumed lengthening of the actin filaments. Sandow [87], Abbott and Ritchie [89] and Guld and Sten-Knudsen [90] investigated the dependence of latency-relaxation on muscle length. They found it with long muscles only (for a detailed review see [91]). Sandow [91] presented the hypothesis that the latency relaxation is caused by release of Ca^2+^ from the sarcoplasmatic reticulum and diffusion to the overlap region. Mulieri [92], in an extensive paper, supported this hypothesis. He found in single fibres a plateau of the latency relaxation curve between 2.8 and 3.2 μm sarcomere length. Its maximum depth (measured at 3 μm) averaged 0.23 % of the maximal tetanus tension and was strongly correlated with the latter. But the discovered plateau was not expected by the original diffusion hypothesis, and the variations above 3.2 μm could not be explained without introducing new features (depression of the latency relaxation by excessive stretch and by stretch-induced distortions of the sarcoplasmatic reticulum).

A glimpse at Figure 5 instantly explains the main details of latency relaxation. When a muscle is stretched before the activation, in order to get it taut, the thin filaments are rotated in counterclockwise direction (see chapter 3.5), and the Z-filaments become twisted to stage E, F or G (Figure 5). When the muscle is now activated, Ca^2+^ is bound on the free sites of the Z-filaments (see chapter 3.7) and produces torque that rotates them from stage G to D and further. This means at first a transient elongation of a few nm by unwinding the distance W, as indicated in stage D of Figure 5, before the contraction by thin filament drilling sets in. The length of the Z-filaments increases from stage G to D and decreases later to A as shown by the length of the bars in Figure 7b. D. K. Hill’s [93] suggestion that latency relaxation is a fall of the thin filament resting tension is fairly correct, since also the Z-filament resting tension of passive muscle should decrease from stage G to A (see chapter 3.5).

The dependence of some components of latency-relaxation was described by Sandow [91]. The amplitude (depth) of it increases from 4% at 32 mm muscle length to 100% at 40 mm muscle length. The elongation should be highest from stage G of the Z-filaments and should decrease from F or E to D. Latency L_1_, the time from the stimulation to onset of positive tension means the untwisting time from stage G to D. It also increases with muscle length from 3.1 ms to 3.8 ms. However, latency L_R_, the time from the stimulation to the onset of relaxation decreases with muscle length from 1.7 ms to 1.2 ms. This may depend on the quicker rise of torque in the shorter cross-arms of stage G or F than in the longer cross-arms of stage E or D (compare the length of the cross-arms in Figure 5).

### 3.5. The Z-filaments in passive muscle

“Active muscle contains an apparently damped element (the contractile component) in series with the undamped elastic one. Resting muscle contains the elastic element, but only to a minor degree the apparently damped element” [39, p. 182]. The non-activated thin filaments (the “contractile component”) of passive muscle lack torque and the Ca^2+^-dependent conformational change of tropomyosin. Their torsional stiffness is diminished [94], but their helical structure is present and must rotate the Z-filaments in counter clockwise direction when a passive muscle is stretched. Yamaguchi *et al.* [58] stretched passive fibres of frog’s anterior tibialis muscle and observed them in the EM before and after the stretch. They found that the structural change of the Z-line from “basket weave” to “small square” occurs around 2.35 μm sarcomere length in intact fibres, and around 3.15 μm sarcomere length in skinned fibres. This transition should occur between the Z-filament twisting stage C and D (see Figure 5).

In the following pages I quote and comment paragraphs from the excellent review article of Proske and Morgan [95] on stretching of passive muscle. D. K. Hill [93] measured the permanent filament resting tension (FRT) on passive frog muscle and an elastic resistance at the beginning of a stretch, the “short range elastic component” (SREC). It increases in hypertonic solutions. “He noted a rise in the permanent resting tension when the SREC increases and suggested that the elastic properties of the SREC were due to the mechanical stiffness of a small number of cross-bridges between actin and myosin in sarcomeres of resting muscle”. Lännergren [96] obtained similar results on the SREC and concluded “that the measured elastic modulus might reflect some component of the muscle fibre other than the cross-bridges.” Moss *et al*. [97] showed “that both mechanically and chemically skinned fibres of frog did not exhibit a SREC in normal relaxing solutions containing low Ca^2+^. However raising Ca^2+^ slightly to levels just below those required for activation, re-established stretch responses typical of those of living passive muscle. It was concluded that one explanation of the data was that Ca^2+^ was required for the development of SREC. There is an increase in SREC in a region of reduced myofilament overlap, followed by a fall at very long lengths [93, 98].” All these facts support the view that the Ca^2+^ sensible Z-filaments (see chapter 3.7) are responsible for the filament resting tension (FRT) and are also twisted during stretch of passive muscle. The SREC may depend on the elastic resistance at the beginning of a stretch, increasing with the twisting stage from A to G and falling when the overlap disappears.. However this resistance should also depend on the viscosity of the medium and on resisting cross-bridges that were indeed later found in relaxed muscle [99,100]. They are also called “weakly bound cross-bridges” [101,102].

Strong tension can arise by stretch of passive muscle. “Bozler [103,104] showed on resting smooth muscles of the snail (*Helix pomatia*) pharynx retractor and on adductor of Pecten that elastic tension was produced when the muscle was stretched. As soon as the length was left constant, the tension dropped and disappeared on an exponential curve…. Bozler concluded that the same viscous factors controlled the tension disappearance as in relaxation from isometric force and that these were situated in the contractile elements” (quot. [105, p. 526]). A. V. Hill [67] did similar stretch experiments on resting and activated tortoise iliotibialis at 0°. The rise of tension after stretch is here not very different between a non-stimulated and stimulated muscle. When the stretch is finished, the tension of the stimulated muscle remains and can increase further, but the tension of the non-stimulated one falls and needs e.g. 10 s to reach a nearly constant level. Such long-lasting after-effects were at first observed by Blix as early as 1893 [106]. After the counter-clockwise rotation during stretch, the tension fall should depend on the slow clockwise back-rotation of the thin filaments, which is less strongly hindered by a small number of cross-bridges than in activated muscle, where many cross-bridges fully prevent the back-rotation. Recent experiments on passive fibres using very quick stretches were done by Bagni *et al.* [107–109]. They concluded that viscous and viscoelastic elements resists against the stretch, perhaps of weakly binding cross-bridges. Such cross-bridges could be responsible for an increased stiffness without a corresponding increase in force and might explain the leading of stiffness over force during tension development, found by Cecchi *et al*. [110].

The question arises, why muscle activation by Ca^2+^ and stretch of passive muscle, both producing increased force? How can a quick stretch substitute the activation [67,118]? Also Linari *et al*. [111] formulated the same problem for insect flight muscle: “The Ca^2+^- and stretch activation are complementary pathways that trigger a common process of cross-bridge attachment and force production.” Hill [39, 40, 67] postulated for the rise of isometric force that the contractile component stretches the series elastic component, and “this is a slow process.” But how can a quickly stretched element, e.g. a spring or a rubber band need 10s or more for relaxation? The solution of this problem is plain. Both processes, Ca^2+^-activation and stretch, produce or increase torque in the filaments. The relaxation of torque occurs by friction and viscosity-retarded rotations, a comparable slow, long-lasting process! The pitfall for the research was that already the 19^th^ century investigators, for example Schwann [112], Ed. Weber [113] and Fick [114], certainly recognized the importance of the elasticity for muscular processes, but perceived the Hookenian elasticity of a stretched spring only. Schwann demonstrated for muscle the quality of an elastic body: Tension-decrease with shortening. Weber tetanized muscles for the first time and found a high elasticity that also decreases with shortening. Heidenhain [115] was an exception. “He concluded correctly that the processes that occur when a muscle is stimulated have a different nature and origin from those that cause a stretched rubber band to shorten” (quot. [68] p. 9). But also Hill and successors overlooked the torsional force and the enormous torsional capacity of the contractile component that also results in the Hookenian elasticity of muscle force.

Stretch of passive muscle changes its metabolism and strongly increases the heat production (“Feng effect”). Clinch assumed release of ionized Ca into the sarcoplasm [68, p. 343–345]. This may depend on ion displacement during winding up of the Z-filaments from stage D to G.

Stretch of passive muscle is very important for a later contraction because it can remove slack. I quote now again the review article of Proske and Morgan [95]. “Passive muscle shows thixotropic behaviour that is contraction and length-dependent changes in response to stretch. At long length a resting muscle will be taut regardless of its previous contraction history. At very short length, it will always be slack [65].” Stretch to long length means passive counter-clockwise rotation of the thin filaments and therefore increased torque in the Z-filaments. Release to short length means clockwise rotation and decrease of torque. “However there is an intermediate length range where a muscle can be either taut or slack depending on the history of contraction and length changes.” The importance of this history was again described already by Blix [106]. “The presence or absence of slack can dramatically alter the shape of the tension rise seen during stretch of a passive muscle. Slack can be introduced at a particular test length by contracting a muscle at a longer length, letting it release completely and then shortening it back to the test length. The slack can be removed by a contraction at the test length.”[95]. In his paper on stretch experiments on passive muscle Hill [67, p. 323] described exactly the procedure for the removement of slack. There are some further experiments in the article of Proske and Morgan [95] which show that the stretch effect is long-lasting. For example, if a stretched muscle is “shortened back to the starting length, slack will develop. Our data suggest that to develop slack fully the muscle must be held at the longer length for several seconds before returning it to the starting length [116].” The higher tension of stretch needs several seconds for relaxation by rotations when hold in the stretched state. When a muscle in a slack state is stretched, the tension rise is delayed and slower as compared with a similarly stretched but taut muscle (see Figure 2 of Proske and Morgan [95]). The slack must at first be taken up by the stretch-produced counter-clockwise thin filament rotation before it can produce further increased torque in the Z-filaments. Campbell and Lakie [117] used pairs of stretches and found that “the initial rise at the beginning of the second stretch was smaller representing the thixotropic nature of the response. During the interval between the two stretches sarcomeres shortened slightly while resting tension rose back towards its original level...”. “Responses of single frog fibres to paired stretches show that in a resting fibre it takes up to 3 minutes following the first stretch, before something approaching the full size of the initial tension rise has recovered [96,117]”. “Something” means here the long-lasting clockwise back-rotation of the thin filaments after the first stretch. It can slightly shorten the sarcomeres when the rotating thin filament helices drill a little against the cross-bridges. The described experiments show that the stretch-produced torque in the Z-filaments of passive muscle is of great importance.

### 3.6. The Ca^2+^-activation

The idea of “activation” and the “active state” set up in muscle by a single stimulus was proposed by Hill [67,118], and discussed in various later papers [68, p. 353]. “...At the end of the latent period there is an abrupt change of state, the contractile component of muscle suddenly becoming capable of bearing a considerable load. The intensity of the active state defined as the maximum force which the muscle can bear without lengthening, was greatest at the start, was maintained for a time and then declined as relaxation sets in.... The decrease of extensibility begins at about the same moment as the heat of activation; the latter is regarded as a product of the chemical process by which the change of mechanical state is effected” [67, p. 320].

“Sten-Knudsen [94] found that the torsional rigidity of an isolated fibre of the frog begins to increase before the main rise of tension, reaching a plateau of about one third the contraction time and maintaining this level until well into the relaxation phase” (quot. A. Huxley [72, p. 301]). The Ca^2+^ addition increases the torsional rigidity of the actin filaments [41,119].

Davies *et al*. [120] “explained the activation heat as due to a release of bound calcium from the sarcoplasmic reticulum. The active state resulting from the increase in free calcium” [105, p. 362]). Ebashi *et al.* [121] suggested “that Ca^2+^ attachment to troponin might cause conformational changes which could be passed on via tropomyosin to F-actin. A modification of the interaction of actin and myosin might then result” [105, p. 325). H. Huxley and Brown [122], H. Huxley [123] “studying changes in low angle equatorial X-ray reflexions, have concluded that the results show a moving out of the centres of mass of the cross-bridges and their attachment to the actin filaments” [105, p. 254]. Recently Zhao and Craig [124] showed by cryo-electron microscopy that on Ca^2+^ binding the helically ordered heads of relaxed filaments become disordered and project further from the filament surface to contact the actin filaments. The process is reversible on removal of Ca^2+^. Full disordering was observed within 30 ms of Ca^2+^ addition, and had started to occur within 10 ms.

Tropomyosin [125] is the main tension-generating device in activated muscle. The tropomyosin molecule is a dimer (length 41 nm) composed of two α-helices wrapped about each other as a coiled-coil. The dimers are connected by head to tail overlap forming a polymer of about l μm length in the two grooves of an actin filament. There are two types of tropomyosin sub-units α and ß. Homodimers (αα) are found in fast or white muscle fibres, heterodimers (αβ) in slow or red fibres [126].

**Figure 8:**
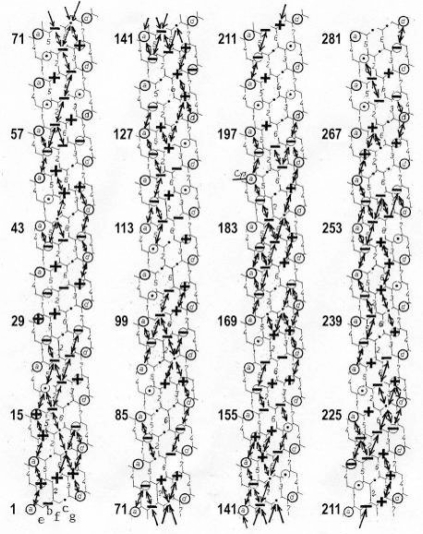
One tropomyosin α-helix is rolled down on a plain. The side-chains l to 284 consist of 40 1/2 groups (heptads) with seven positions a, b, c, d, e, f and g. The positions a and d form strong hydrophobic bonds with the positions d and a of the second α-helix. The two α-helices twist around one another to form a coiled-coil. The strongly charged side-chains on the coiled-coil surface are drawn as + and −. Coulombic repulsion between equally charged side-chains arranged in axial direction and, according to the “interaction coefficients”, is indicated by double arrows. The H-bonds of the α-helix of one heptad are numbered l to 7. Particularly the outer H-bonds 2, 3, 5 and 6 are stretched by the Coulombic repulsion of the charged side-chains. Every side-chain discharge should shorten the outer H-bonds cooperatively, inevitably producing torque (see Figure 9).

**Figure 9:**
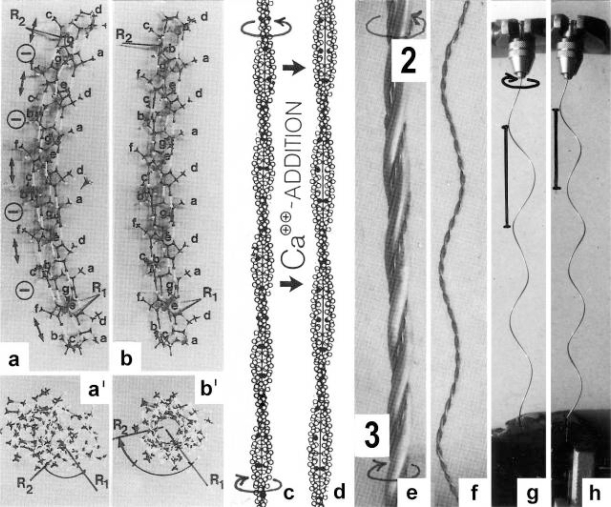
The conformational change of tropomyosin. (a) Molecule model of a tropomyosin α-helix with indicated Coulombic repulsion between negatively charged side-chains (double arrows), that stretch the outer H-bonds. (b) The curvature of the α-helix decreases when the repulsion is diminished by side-chain discharge. (a’) and (b’) The angle between two rods R_1_ and R_2_ increases, i.e. torque is produced when the H-bonds shorten, that causes rotation (arrows in (c)), and a conformational change of the left-handed coiled-coil superhelix shown in (d). (e) A tropomyosin coiled-coil is situated in each groove of the right-handed actin double helix which twists the actin filament (arrows). (f) One coiled-coil is unwound from the actin helix model. (g) Twisting a steel wire helix in the direction of the arrow increases the helix pitch. The relaxed steel helix is shown in (h). Such a pitch change of tropomyosin is not possible since it is bound in the groove of the solid actin filament. It can show the “tropomyosin shift” only (see Figure 2a).

Tropomyosin is one of the most charged proteins. There are 284 amino acids [127]. 136 are strongly charged, 81 are negative and 55 are positive. They are arranged mainly on the outer, convex side of the α-helix. Nearly all of the 82 side-chains arranged at the inner concave side of the α-helix are hydrophobic, connecting the two helices strongly together by hydrophobic side-chain interlocking (“knops into holes” according to Crick [128,129]). “The biological significance of the coiled-coil is evident: this structure confers rigidity to highly charged polypeptide chains in an aqueous environment. In contrast single chain synthetic copolymers of lysin and glutamic acid have little α-helix under condition of neutral pH, where the two-stranded coiled-coil is stable [130]” (quoted from [131]). However the question arises, to what purpose is the surface of the tropomyosin coiled-coil so strongly charged? According to Pauling and Corey [132], and Corey and Pauling [133], the interaction of side-chains can influence the length of the α-helical H-bonds. Also Bailey [134] contemplating electrostatic contraction theories noticed that any modification of the electrostatic side chain forces must work in relation to the H-bonding of the α-helix, and Morales [135] still emphasing the importance of the ion-polyelectrolyte effect, said: “Nothing...has weakened my intuitive conviction that the tension-generating device in excited muscle will prove to be a mechanically continuous structure... nor am I ready to relinquish my faith in Coulombic interactions as the most rapidly generated and the most long-ranges of the forces that the transducer could employ” (quoted from [105, p. 181]). Thus it seems [22,24] that equally charged side chains arranged in axial direction repel each other and stretch so the axially directed α-helical H-bonds, which are arranged below the side chains on the outer surface of the coiled-coil. The H-bonds are numbered l to 7 in Figure 8 that shows one α-helix rolled down on a plain. The shorter H-bonds l, 4 and 7 near the coiled-coil axis, are protected because of the strong hydrophobic interactions between the side-chains on the positions a and d. This behaviour has the effect that the tropomyosin coiled-coil can change its pitch and conformation (see Figure 9c, d) without changing its axial length [24].

In order to comprehend whether side-chains repel or attract, we can use the “interaction coefficients” introduced by Kuntz *et al*. [136]. The repulsion between strongly charged side-chains Glu^−^ and Asp^−^, or Lys^+^ and Arg^+^, is here expressed by the positive number +25, while the attraction between Glu^−^ or Asp^−^ and Lys^+^ or Arg^+^, is expressed by the negative number −10. Lys^+^ or Glu^−^ and Leu repel with +15, Lys^+^ or Asp^−^ and Met repel with +10, etc. All positions of side-chains that can repel each other according to the interaction coefficients which can stretch the H-bonds when arranged in axial direction are indicated by double arrows in Figure 8. But not all repelling forces stretch the H-bonds. Repelling forces that work in perpendicular direction to the helix axis should not influence the H-bond length. Attractive forces may shorten H-bonds, if they can form salt-bridges (neutralization). Attraction only (without neutralization) should weaken the repelling force against another equally charged side-chain. The same weakening effect of an attractive force should be possible also between neighbouring side-chains in the axial direction, for example between b–e or c–f (the side-chain positions are indicated in Figure 8, bottom, left). Since the positively charged side-chains are longer than the negatively charged ones, the attraction in the axial direction, for example b–f, would not shorten the hydrogen bonds directly by the attraction, since they attract not more in axial direction [22].

But they may indirectly weaken the repulsion against another side-chain. In spite of these uncertainties the sums of the interaction coefficients which describe the forces between side-chains that work in axial direction yield a surplus of the repelling forces that cooperatively can stretch the H-bonds. The cooperativity in an α-helix means that the H-bond length is effective for other H-bonds on the coiled-coil surface. An H-bond is also an electrostatic attraction between a positive proton and two negatively charged atoms. If a charged side-chain can interact with the carbonyl and imino groups of the amide groups, as was assumed by Corey and Pauling [133, p. 29, 30], a direct influence on the H-bond lengths could be possible. The effect of the predominantly negative side-chains may be better described as net charge of the tropomyosin molecule, that works against the attractive H-bond system below, and may stretch it as an unity. The positively charged side-chains may weaken this net charge, but are important for stabilization. Negative side-chains only would destabilize the molecule. Gaffin *et al.* [137] found that the “net charge of specific sites on TM might be a major determinant of its role in modulating cardiac muscle performance”.

The surplus of the repelling forces over the attractive forces of the side-chains means that all outer H-bonds of a tropomyosin coiled-coil α-helix are stretched and increase the curvature of this α-helix, as shown by a Minit-model with stretched outer H-bonds 2, 3, 5 and 6 of about 2.95 å (Figure 9a, from [24]). The inner H-bonds 1, 4 and 7 have a normal length of 2.67 å. The repelling forces between negative side-chains are indicated by double arrows. A discharge of these side-chains by interaction with positively charged molecules would diminish the repelling forces and decreases the curvature of the helix. Figure 9b shows the same model with outer H-bond length of about 2.70 å and unchanged length (2.67 å) of the inner H-bonds. H-bond shortening means pitch decrease and twist increase of the α-helix [22–24,139]. The twist-increase is demonstrated by the angle-increase between two rods (R1 and R2) perpendicularly arranged to the helix axis at a distance of 27 residues (Figure 9a’, b’). The resulting conformational change of the whole tropomyosin coiled-coil is approximately shown in Figure 9c, d. The pitch-increase of the left-handed coiled-coil superhelix in Figure 9d is combined with the generation of torque that accumulates in long filaments and can produce counter-directed rotations of the coiled-coil ends (arrows in Figure 9c). The amount of the coiled-coil pitch-increase and torque generation depends on the intensity of the activation. The coiled-coil pitch is very sensible against changes of the α-helix pitch and twist [132,139,140: Figure 15.32). The coiled-coil pitch of tropomyosin was described by diverse authors with different values. Carolyn Cohen [141] pointed at first to its variability. During activation, it may change in the range between 11 and 19 nm [22].

Interactions with the highly charged tropomyosin surface are possible with molecules of the medium, with the actin filament, with troponin and with myosin. Best known is the interaction with troponin [142,143, for recent work see 144]. The troponin complex has a globular and rod like domain. The head containing TnC and TnI binds near residues 150–180 of tropomyosin (see Figure 8). After Ca^2+^ binding to TnC the positively charged helical tail, containing TnT1 and TnT2 shows extensive interactions in the range near the Cys-position 190 of tropomyosin to beyond the COOH terminus. It is probably that this interaction is responsible for a main part of the tropomyosin’s conformational change.

One tropomyosin coiled-coil is situated in each groove of the right-handed double-helical actin filament, as shown by the model in Figure 9e. There is a hierarchy of helices: The first order is the right-handed α-helix. The second order is the left-handed coiled-coil helix (Figure 9c, d), and the third order helix in the groove of the actin filament is again right-handed. A model of this helix is shown unwound in Figure 9f. According to Brown *et al*. [145], it is the high alanin content in the molecule core that gives rise to specific bends in the tropomyosin axis by an axial staggering of the two α-helices. The conformational change of tropomyosin should change the shape of the third order helix too, if it would be free. But such a change is not known, since both tropomyosin coiled-coils are fastened, being wound about the actin filament, and are bound to it, at least over troponin in distances of 38.5 nm. We can only see, that a right-handed steel-helix which is strongly twisted in clockwise direction (arrow in Figure 9g, the other end is fixed), shows an increased helical pitch. Relaxation, e.g. by clockwise rotation of the other end, decreases the pitch again (Figure 9h). Thus the conformational change may be recognizable as the “tropomyosin-shift” only [146–148]. Recent EM-work by Lehman *et al*. [149] and Vibert *et al*. [35] has shown, that the two strands associated in the nonactivated state with the outer domain of actin (A_0_ in Figure 2a) move across the actin surface closer to the inner domain (A_1_ in Figure 2a). Besides Ca^2+^, the tropomyosin motion takes place after decoration of the actin filament with myosin S1. Tropomyosin was located in the same A_i_ position “whether or not Ca2+ is present ... hence in the absence of ATP, the binding of S1 is sufficient to move tropomyosin” [35]. Activation of contraction by strongly attached myosin cross-bridges in the absence of Ca^2+^ was shown [e. g. 150,151]. Side chains of S1 may directly interact with the highly charged tropomyosin surface and could produce H-bond shortening.

It was demonstrated by the α-helix model with stretched outer H-bonds, 3.00 å long, and inner H-bonds, 2.67 å long, that shortening of the outer H-bonds for example to 2.71 å, produces an angle-difference between R1 and R2 of 25° (Figure 9a’, b’). This means for a tropomyosin length of about 1 μm in the thin filaments of skeletal muscle, a torsional force for about 17 rotations of the free ends [22]. As recently found by using fluorescence energy transfer data (FRET), Ca^2+^ causes not only azimuthal motions of tropomyosin in the grooves of F-actin but also rotations around the coiled-coil axis, which can lead to a rolling motion on the actin filament surface [25]. The rolling motion yields other regions of the tropomyosin surface in close contact with the actin filament, and thus may cause further interactions.

The torque produced by the whole tropomyosin is cooperatively transmitted to the actin filament and must twist it, so that the free distal end of the thin filament can rotate (arrow 3 in Figure 9e and Figure 1a). The rotation of the proximal end (arrow 2) is restricted, since the torque is equilibrated by the torque of the anchoring Z-filaments (arrow 1).

The torsional compliance of the actin filament (comp. [41] and the papers of Egelman, quoted e.g. in [152]) is very important for filament sliding in muscle and *in vitro*. A rigid actin filament would not take over the torque of tropomyosin and would not rotate. Moreover the stable actin filament is an important support for the two torque-producing tropomyosin coiled-coils. Without this support the torque would produce self intertwining (supercoiling) of the coiled-coils like a twisted telephone cord. The rotational activity would remain in the molecular level.

What further occurs with the thin filaments, after having taken over the torsional rotation of tropomyosin (or myosin); depends substantially on the load of muscle: The thin filament helices (the “contractile component”) exactly their distal ends, can translocate (= drill, arrow 4 in Figure 1a) in clockwise direction (arrow 3) into the myosin cross-bridges, as already described in the first chapter. The state after the contraction is shown in Figure 1b. The velocity of this isotonic shortening is load-dependent (see chapter 3.9). With a too heavy load, or when the muscle is clamped by both ends, the drilling rotation (arrow 3) is prevented, and the rotation of the other end (arrow 2) can work against the torque of the anchoring twisted Z-filaments (the “series elastic component”). This means the rise of the isometric tension from Figure 1a to Figure 1c by untwisting the Z-filaments. The full isometric state shown in Figure 1c is ready for shortening by drilling of the thin filaments (arrows 3 and 4) when released.

The tropomyosin shift and its hindered conformational change should produce increasing internal torque and a shape change that strengthens the second actin layer line in X-ray diagrams, an effect also observed when the muscle fibres are stretched beyond filament overlap [153]. In the time course of the sequential changes of X-ray reflections from frog sartorius muscle during the rise of isometric tension [153–155] the earliest change is the intensity increase of the second actin layer line A2. It is thought as indicative for the tropomyosin shift. 10 to 15 ms later follows the intensity increase of the equatorial reflections indicative for myosin S1 movement towards actin, and still later the increase of isometric tension. The tropomyosin motion should be connected with torque generation in the thin filament that can change in part also the actin structure, since the intensity of the first actin layer line A1 (365 å) decreases strongly.

The binding of Ca^2+^ to troponin that results in shortening of the outer H-bonds of the tropomyosin coiled-coils causes a conformational change as shown in Figure 9 from a to b and from c to d. However that conformational change does not occur instantly, because the tropomyosin coiled-coils are bound and wound about the actin filament. The produced torque twists the actin filament, but the resulting rotation of the thin filament (arrow 3 in Figure 1) is retarded by friction and viscosity. Therefore the conformational change, recognizable as the tropomyosin shift, is also retarded and needs about 35 ms to reach its maximum [153, 156].

Why is the rise of the isometric force the last in the time course and 25–30 ms delayed as compared with the tropomyosin shift? The answer may be: Muscle force needs clockwise translocation or drilling rotation of the thin filaments (arrows 3 and 4 in Figure 1). This motion depends on a higher number of myosin cross-bridges that arrive slowly [154]. There is a mass transfer from the thick to the thin filaments during the rise of isometric tension. Is there also a winding up of S1 substance? Become the thin filaments thicker, as Tsukita and Yano [100] describe after electron microscopic observations? Why the myosin heads labelling actin layer lines disappear in X-ray diagrams, when ATP is added (Yagi, in [2, p. 389])? In any case, the mass transfer seems necessary for lifting a heavy load and for the isometric force. It is not appropriate for unloaded shortening or lifting a small load. During full isometric force many cross-bridges must be in close contact with the helical surface of the rotating thin filament. Otherwise the kinematics of the rotating actin helix would change. The cross-bridges would slip and the helical turns would move as helical waves into the opposite direction. Thus the time-lag of isometric force may be based on initial quicker non-drilling rotation caused by the small number of cross-bridges which slip and do not resist but may already increase the stiffness. Weber and Hasselbach [157] discovered that in hydrolysis of ATP by muscle fibrils, there was an explosive phase lasting some 15 s, during which the ATP was split at least twice as fast as during the steady phase. This and following papers on an initial burst of phosphate liberation were discussed by Needham [105, p. 280 ff.]. Cecchi *et al*. [158] conclude from their measurements (see Figure 11d) that at the beginning of an isometric tetanus the steady force for a given velocity (less than V_max_)increases with time, i.e. the number of cross-bridges increases. He *et al*. [159] described increased ATP-splitting at the start of isometric contraction of skinned fibres, and Josephson and Edman [160] found at the start of the tetanic stimulation V_0_ was about 30 % greater than during the plateau of the tetanus.

It was originally proposed by Harrington [161–163] “that melting and shortening of α-helical structures in the myosin S2 region contribute to muscle force generation. In support of this hypothesis, polyclonal anti S2 antibody has been shown to reduce Ca^2+^-activated isometric force in glycerinated skeletal muscle fibres, but ATPase activity of the fibres and the unloaded shortening velocity of isolated myofibrils undergo little change [164,165]. Furthermore, Sugi *et al*. [166] demonstrated that in glycerinated skeletal muscle fibres, Ca^2+^-activated isometric force and muscle fibre stiffness are uncoupled from Mg-ATPase activity in the presence of anti S2 antibody, strongly suggesting an essential role of the S2 region in muscle contraction” [quotation from 167].

The tails of the myosin subfilaments are similar coiled-coils as tropomyosin. During activation they project out of their compact helically ordered arrangement in the thick filaments as was shown by Zhao and Craig [124] on scallop thick filaments after Ca^2+^ activation. Probably there is a similar process of torque generation in the myosin tails as described for the tropomyosin coiled-coils that enable them to drill out and contact the thin filaments. The amount of this myosin motion may be limited to a few rotations.

When torque is generated, its cooperative transfer to the heads should be interrupted by the antibody S2. Decreased or eliminated torque can impair the drilling out process of the cross-bridges, reduce their number arriving at the thin filaments, but may also change their particular contact with the thin filaments. As described above, the cross-bridge number and the amount of their pressure on the rotating thin filament helix should determine the kinematics of this motion: Translocation by drilling or “idle rotation”. Winding up of S1 chains and following unwinding with ATP-splitting, could still occur during “idle rotation” of the thin filaments. The force transmission from myosin to actin by winding up and unwinding may be disturbed too.

### 3.7. The binding dynamics of the Z-filaments for Ca^2+^

**Figure 10:**
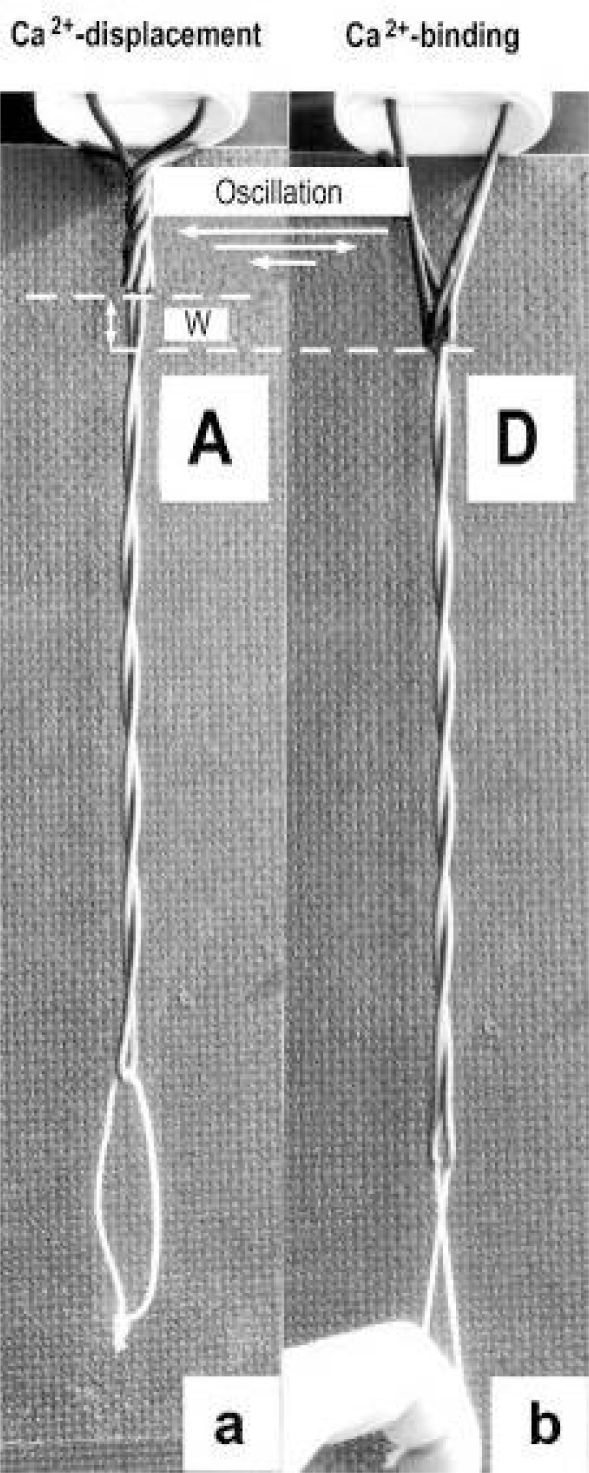
A rigid wire-helix model with Z-filaments in the A stage (a) is strongly stretched to the D stage (b). This means for the α-actinin Z-filaments of muscle that Ca^2+^ is bound by the liberated sites during stretch in (b), and displaced as “extra Ca^2+^” when again relaxed in (a). Since Ca^2+^ binding produces more torque in the Z-filaments, feedback-regulated damped oscillations of the Z-filaments can appear (arrows).

The Z-filaments are strong rod-like fibres of α-actinin (length about 35 nm) that connect the thin filaments of neighbouring sarcomeres. α-Actinin is a protein of the spectrin family. It forms complex triple-stranded α-helical coiled-coils with C-terminal EF-hands as the Ca^2+^ binding units [168–170]. Since Ca^2+^ is bound to the surface of the Z-filaments, some Ca^2+^ is displaced from the filament region that is wound up as indicated in Figure 10. Therefore the sensitivity of myofilaments to Ca^2+^ changes with muscle length because during muscle stretch the thin filaments rotate passively in counterclockwise direction and change the Z-filament twisting stage from A to D. It was first shown by Endo [171] on skinned amphibian fibres, when the sarcomere length was increased from 2.0 μm to 2.9 μm that the threshold for contraction shifted towards a lower Ca^2+^ concentration by a factor of 1.4. Later work demonstrated that the entire force-pCa curve is shifted [172 for a review]. However, Balnave and Allen [173] reported for single fibres of mouse muscle, that while at moderate long length Ca^2+^ sensitivity increases above the level at optimum, stretching the muscle further led to a fall in Ca^2+^ sensitivity [quot. 95]. Obviously this is the result of further stretch by twisting up the Z-filaments from stage D to G. Thus the “series elastic component” is not fully passive as was postulated by the original “two component model”. This wrong assumption was one of the reasons that led Woledge *et al*. [49, p. 37] to abandon the model.

The binding dynamics of the Z-filaments for Ca^2+^ and its displacement as “extra Ca^2+^” is beautifully shown by quick release and stretch experiments using aequorin luminescence as an indicator for free Ca^2+^-ions [174–176]. The curve of the displaced “extra Ca^2+^” (“extra light”) is positive after a quick release from about stage C to B. It is negative after stretch from about B to D, and is biphasic, at first positive and then negative, when the stretch takes place from about D to F or G, and the tension then again drops to D. The curve of “extra Ca^2+^” mirrors exactly the expected displacement or binding of Ca^2+^ after changing the actual twisting stage.

The displacement of Ca^2+^ from D to A means torque decrease, reversal of the rotational direction, and again unwinding of the Z-filaments. Now more Ca^2+^ can be bound and produces once more winding up. So feedback-regulated damped oscillations appear that are indicated by arrows in Figure 10. These oscillations are typical for quick releases of high amplitude, e.g. in Figure 15a, and are also shown as velocity oscillations during isotonic shortening after quick load decrease (Figure 23b).

The force change after quick stretch or release by Ca^2+^ binding or displacement is also expressed by the Huxley-Simmons phase 3 (Figure 22, see chapter 3.14). A reviewer doubts the regulatoric binding of Ca to the Z-filaments. But I sum up: (1) The Z-filaments consist of α-actinin that contains E-F-hands. (2) The dependence of the Ca-sensitivity on mucle length has been repeatedly described. (3) An alteration of muscle length by stretch changes the twisting stage of the four Z-filaments. (4) Most Ca is bound to the four Z-filaments when fully unwound in the isometric stage D. The amount of the displaced “extra-Ca” increases always with the winding up-stage of the four Z-filaments.

### 3.8. The “Fenn-effect”

Fenn [177, 178] found during a twitch or short tetanus, when the muscle lifted a weight that the increase in heat production was roughly proportional to the work done. An extra amount of energy was mobilized which was not found on isometric contraction. Energy is mobilized as needed. “Energy liberated by contraction of a single muscle fibre for a given stimulation is not dependent solely upon the initial mechanical and physiological conditions of the muscle, but can be modified by the nature of the load which the muscle discovers it must lift” [177]. Fenn had predecessors in his work, and he referred to them in some detail in his introduction. Heidenhain [115] found “that when the initial tension was altered in an isometric contraction, or the work was altered by changing the load, the total energy changed too; that the muscle contained a “governor” by which the energy used was largely determined by length and load” [68, p. 9]. Similar results were found by Fick [114] who introduced the terms “isotonic” and “isometric”. He “asked himself the question whether upon isometric stimulation a full store of potential energy was produced ready for mechanical performance under the right conditions. To test this point he held the muscle after stimulation till maximum tension was developed and then released it to shorten under load. More heat was developed in the isotonic than in the corresponding isometric contraction” [105, p. 55].

It was shown by Kushmerick and Davies [45] that ATP cleavage was correlated with performance of work over a range of velocities. “The chemical change (and thus the explained energy) behaves like the work and not like the energy (heat + work) production, which remains quite high because of the high rate of heat production during rapid shortening [179]”. “An unidentified process was shown by Curtin *et al*. [46] to be occurring during working contractions and contributing to the production of energy”. “However, there was enough ATP splitting occurring to explain all the work” (quot. from [49, p. 261]). Presumably the “unidentified process” is the thin filament drilling rotation that produces frictional heat of shortening (see chapter 3.11).

The Fenn-effect is a simple consequence of the ingenious construction of muscle. The anchorage of each thin filament by four Z-filaments means, that the amount of muscle load or force determines a certain twisting stage of the four Z-filaments, as shown by the model of Figure 4. Thus the muscle needs no “Governor”, and the load is not “discovered” by the muscle. It is automatically balanced by the suitable twisting stage and tension of the series elastic Z-filaments. Since the amount of Ca^2+^ bound to the free surface of the Z-filaments increases from stage A to the isometric stage D (chapter 3.7), torque and tension increases as well and promotes work and energy liberation.

### 3.9. The force-velocity relation

**Figure 11:**
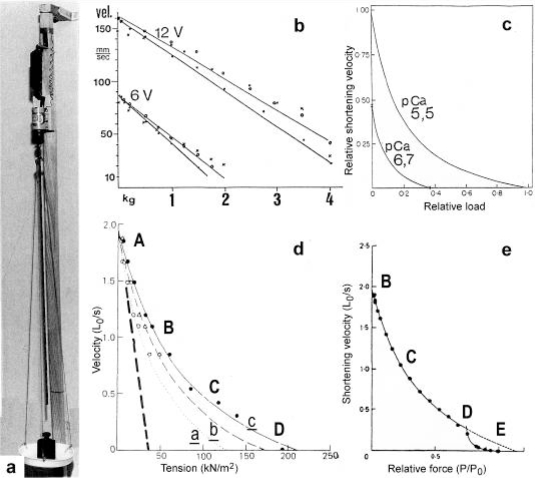
The force-velocity relation. (a) A long electromotor-driven rotating screw-coil (well lubricated) lifts its nut with a plate for weights. (b) Its force-velocity relations are straight lines that depend on the electromotoric force 6V or 12V, as shown (from Jarosch [74]). (c) The force-velocity relations of muscle are hyperbolae that depend on the Ca^2+^ concentration (from Julian *et al.* [181]; with permission). (d) The curvature of the curves indicated with a, b and c increases with time at the beginning of an isometric tetanus (curve a: 0.32 P_0_, 75 ms after the first stimulation, curve b: 0.50 P_0_ after 100 ms, curve c: l.00 P_0_, after 190 ms). The steady force for a given velocity increases with time (from Cecchi *et al.* [158]; with permission). The force-velocity of muscle would be linear (as the curves in (b), or the added dashed line in (d), or the velocity curves in Figure 23), when only the load would increase, but the torsional force would remain constant. Since also the torsional driving force increases from Z-filament twisting stage A to D, as indicated, the relation is a hyperbola. (e) Deviation of the force-velocity curve from an exact hyperbola in the high force region (from Edman *et al.* [185]; with permission). Here, all twisting stages may be shifted so, that stage D is already reached by a slow drilling velocity.

A basic concept of this work is that isotonic shortening is a M-band directed drilling rotation of the thin filaments. This means shortening of the sarcomeres and lifting of the muscle load. Thus lifting of the load is the consequence of the thin filament drilling against the myosin cross-bridges that function as a nut-coil. Figure 11a [from 74] shows a well lubricated electromotor-driven screw-coil that lifts its nut together with a plate, where different weights can be imposed. The force-velocity relation of this rotating screw is shown in Figure 11b. Since the electromotor force increases with the voltage number, measurements of the lifting velocity of different loads were performed at 6 V and 12 V. The results are straight lines, i.e. the relation between force and velocity is linear. The force is here constant and the load is variable. The velocity decreases with increasing load, as it is the case with the steady state shortening curves of muscle in Figure 23. Analogous to the electromotoric force, the force-velocity relation of muscle depends on the Ca^2+^ concentration (Figure 11c, from [181]), but the curves are nonlinear and are parts of a rectangular hyperbola [39] (Figure 11c, d, e). Why is the force-velocity curve of muscle non-linear? The muscle force-velocity curve passes all Z-filament twisting stages from A to D as indicated in Figure 11d. That means exponential torque-increase from stage A to D together with the load, as it is shown by the model of Figure 4. Increasing load means not only more tensile stress but also more torque. The curve of a constant force with variable load is approximately indicated by the thick dashed line in Figure 11d. Cecchi *et al*. [158] observed the force-velocity relation during rising force at three different times (Figure 11d). Curve a, b and c refer to releases beginning 75, 100 and 190 ms after the first stimulation. The tension had already developed to 0.32 P_0_, 0.50 P_0_ and 1.00 P_0_. The curves move to the right with increasing tension and torque. The authors conclude “that at the beginning of an isometric tetanus the steady force for a given velocity (less than V_max_) increases with time” (for more details see chapter 3.6). Also the force-extension curve of muscle depends on the exponentially decreasing force of the Z-filaments from stage D to A.

It was found by Edman *et al*. [182] that the force-velocity curve could deviate from an exact hyperbola. Similar deviations were described by Lännergren [183,184]. The deviations are striking with external loads above 0.8 P_0_ (Figure 11e, from [185]). This behaviour may be explained by assuming that the Z-filament stage D is reached before the shortening velocity is zero. All the twisting stages may be displaced into the direction of the quicker velocity, as indicated in Figure 11e. This agrees with the further observation of Edman [186], that the rate of stiffness increase with increasing load becomes suddenly much larger above 0.8 P_0_. The passage from the right-handed to left-handed twisting stage of the Z-filaments in stage D is connected at the model of Figure 4 with a sudden increase of the resistance against stretch (“point of force reversal” in Figure 5). The gentle unwinding from stage A to D occurs into the same direction as the pull of stretch. Winding up from stage D to G occurs into the opposite direction to the pull with much more resistance.

### 3.10. The length-tension relation and the “unexplained energy” in isometric contraction

The dependence of muscle force on muscle length (sarcomere length) was at first measured on frog muscle by Ramsey and Street [61]. They stretched the relaxed muscle to the desired length and measured the force after activation. The resulting curve was improved by considering the sarcomere-length non-uniformities [187, 188] and confirmed by other authors. It consists of an ascending limb with increasing force from about 1 to 2.00 μm sarcomere length, a plateau with maximum isometric force (2.00 μm to 2.25 μm sarcomere slack length), and the descending limb from 2.25 μm to about 3.65 μm sarcomere length. At 3.65 μm there is not more overlap. Mammalian muscles show little longer thin filaments than frog, so that the zero force is found at about 4.0 μm sarcomere length [189, 49]. The upper part of a frog tension length curve is shown in Figure 12 from [190].

Gordon *et al*. [188] explained the decreased force in the ascending limb by the overlap of the thin filaments with each other and by the collision of the thick filaments with the Z-lines. A contraction with a large sliding distance can also lead to short sarcomeres of the ascending limb. This could be connected with torque decrease and force depression (force deficit). Incomplete activation at short sarcomere length is a further explanation for the reduced force [191].

Since the descending limb of the length-tension curve depends on the amount of overlap between thick and thin filaments and thus on the number of the connecting myosin cross-bridges, the idea that these cross-bridges are “independent force generators” was advanced [5, 238]. This conclusion was further supported by the quick release experiments of Huxley and Simmons [192, 226], which showed that both, the T_1_ and T_2_ tension curves scaled down in direct proportion to the amount of overlap (Figure 16d). However, according to the rotation model this interpretation is wrong. The steady isometric state depends on a balance between the thin filament torque and the resisting force of the cross-bridges. When the number of the resisting cross-bridges is reduced by the decreased overlap, they cannot hold the full torque of the thin filaments since they are not iron nut-coils for the thin filament helices. They slip, and the thin filaments can rotate without translocation (“idle rotation”), and reduce their torque as long as it is equilibrated by the resisting force of the reduced number of cross-bridges. So the decline of the isometric force above 2.25 μm sarcomere length of the descending limb is not the consequence of a reduced number of “independent force generators” but the effect of a reduced torque in the thin filaments that results by the decreased number of cross-bridges [50]. The strongest torque can be produced in the short sarcomeres of slack length. “Muscles are rather quicker in shorter length” [68, p. 142].

Slipping of cross-bridges means their passive cycling [138], (for a comb model of cycling cross-bridges see [193]) that should produce heat by friction as an “unexplained energy”. When at the beginning of isometric contraction the thin filament torque is stronger than the force of the resisting cross-bridges the thin filaments can rotate during some time, and the cross-bridges must cycle passively. Already Bugnard [194] has shown that a periodically tetanized muscle attains a steady state in which the amount of heat produced during each successive contraction (initial heat) is the same, but less then that produced during the first contraction of the series. According to Gilbert *et al*. [195] much unexplained energy is produced in the first 2 s of isometric contraction. After Homsher *et al*. [196] it is only found during the first 5–8 s of stimulation in a 20 s tetanus, but not later. Also in long sarcomeres with reduced filament overlap a significant amount of unexplained energy is produced, but its amount is smaller than in short sarcomeres [180]. Paul [197] tetanized frog sartorius muscle for 3 s every 256 s and measured the steady state rate of oxygen consumption, the steady state rate of heat production, the initial heat production and the amount of high energy phosphates consumed during the contraction. The calculated enthalpy showed an energy balance for the complete contraction-relaxation-recovery cycle, but a significant amount of unexplained enthalpy during the initial phase of a steady-state tetanus and in a contraction after a long rest (all quotations from [49]).

I gratefully acknowledge a reviewer’s indication to the recently puslished papers of Shimamoto *et al*. [198, 199] which showed that the isometric length-tension relationship is not proportional to the overlap between thick and thin filaments under intermediate activation conditions. As described above, the steady isometric state during full activation depends on a balance between the thin filament torque and the resisting force of the cross-bridges. When the torque is reduced, e.g. by lower Ca-activation, the balance is not reached. A decrease of the overlap by stretch means therefore no or a smaller force reduction than under full activating conditions.

### 3.11. Unexplained shortening heat is produced by the friction of drilling

“The rate of heat production rises rapidly to its maximum about half way through the latent period” [67, 118, 200]. Hill differentiated activation heat, shortening heat and maintenance heat. The reason for the heat-production was for a long time supposed to be derived only from chemical processes. But there was found unexplained heat production [195] or “unexplained enthalpy” which is proportional to filament overlap [201]. Hill [39, p. 178/79] found that the heat rate of “isometric short” was rather greater than “isometric long” (“short” and “long” mean the muscle sarcomere length with much and little overlap, respectively). It was already discussed by Fick [114], Hill and Hartree [202], Hill [203], Lupton [204] and Wyman [205] that the mechanical energy of muscle not used for its work, is degraded into heat by viscous and frictional resistance. “The shortening heat can be expressed as though it were derived from the work done in shortening a distance x cm against a frictional resistance of **a** g wt” ([39, p. 160], **a** is the dynamic constant of Hill’s “characteristic equation”). “In shortening a distance x cm, extra heat **a** x g cm is set free” [39, p. 161]. Hill found later [118] that the larger the initial muscle length the greater the distance shortened and the greater the heat produced. Hill [39, p. 168/69) found further during relaxation: “When the muscle lengthens under load, the energy of load (the work done by the muscle on it) appears as heat: hence the rapid upstroke (of heat-curve C in Hill’s Figure 8) during relaxation”. “The facts are really quite simple: the energy of the load, and the energy of any stretched bodies in series with the muscle, is turned straight into heat when the muscle relaxes”. In accordance with these results I supposed [50] that the rotation of the thin filaments must produce friction and frictional heat when they drill against closely adjacent cross-bridges, and that the constant **a** may appear as a rate of friction. The single actin monomers seen as “papillae” in the grooves of the actin filament (Figure 2a, b) should produce friction and frictional heat. Increased load should increase both along these “papillae”. Increasing torsional pressure of the myosin heads upon the rotating thin filaments should have the same effect. Hill [179] found increasing shortening heat with increasing load on frog muscles. But little or no shortening heat is produced in certain muscles, for example in cardiac muscles [206]. The lack of shortening heat in cardiac muscles could depend on a to and fro sliding by periodic winding up and unwinding of myosin chains during the beating cycle. This could produce less friction since the frictional resistance of the cross-bridge heads decreases when its substance is wound up and drills together with the thin filaments. ATP may be split during unwinding. After an x-ray study on dog cardiac muscle Matsubara *et al.* [207] conclude: There is a “slow transfer of myosin heads in heart muscle...the myosin as a whole never go into the “quiescent state”, there is always a considerable number of heads in the vicinity of the thin filament during the diastolic phase”. According to Suga [208, p. 310] the series elastic component of cardiac muscle is stretched by 7 % of muscle length. That is much more than the 1–2 % of skeletal muscle. This means that the sliding distance of cardiac muscle corresponds with the contraction amplitude of the series elastic component. The same may be valid for the high-frequency oscillation of insect flight muscles.

Pollack and co-workers [e. g. 209, 210] described stepwise shortening during the sliding process in different muscles. Recently, they found with a highly developed techniques with single myofibrils “that the essential event of muscle shortening (and lengthening) is a step whose rise is an integer multiple of 2.7 nm...that is equal to the linear repeat of actin monomers” [211]. According to Yakovenko *et al*. [212] and Liu and Pollack [213], the most numerous step-size obtained from records from single myofibrillar sarcomeres is 5.4 nm. This is the double value of 2.7 nm and the distance between the “papillae” in the grooves of actin filament in Figure 2a, b. Moreover the histogram peaks are broader in passive fibres than those in active fibres, and the minimal step size of passive fibres was smaller, ranging from 2.0 to 2.3 nm [211]. The axial distance between the friction producing monomers in passive fibres seems to be smaller and less exactly determined than in activated fibres.

The heat of friction may be produced when the thin filaments drill into the cross-bridges. If the latter are not wound up they can snap from cleft to cleft between the actin monomers or may jump over them.

### 3.12. Unloaded shortening velocity and sarcomere length

**Figure 12:**
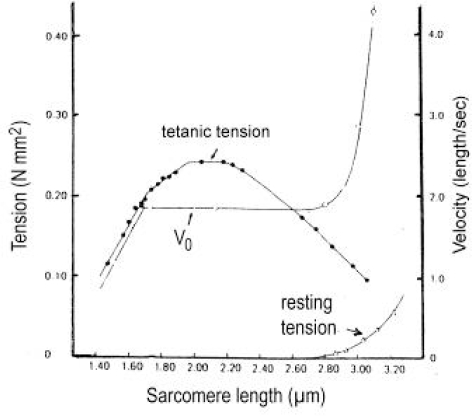
Upper part of the sarcomere length-tension relation and the dependence of the unloaded shortening velocity (curve V_0_) on sarcomere length (from Edman [190]; with permission). For more details see text.

Edman [190] measured the unloaded shortening velocity with the slack-test method described by Hill [214]. As shown in Figure 12, he found striking velocity differences in dependence of sarcomere length (curve V_0_). In the range of sarcomere lengths l.65–2.7 μm, V_0_ was constant. At 1.65 μm sarcomere length, it decreases abruptly. At sarcomere lengths longer than 2.7 μm, V_0_ increases at first smoothly and then quickly with the sarcomere length.

**Figure 13:**
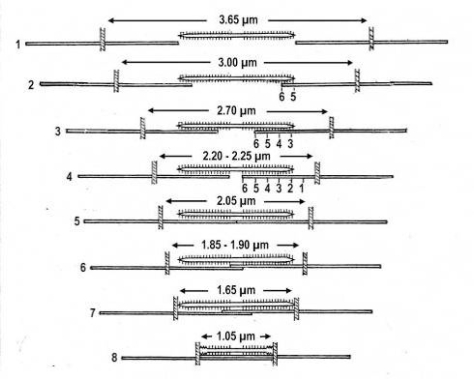
Extended diagram of Gordon *et al*. [188] on the relation of sarcomere length and shortening velocity. The strong increase of the unloaded shortening velocity at sarcomere length 2.7 μm (see Figure 12), can be explained not only by the increase of the resting tension in this region, but also by the contact of the quicker rotating thin filament tips with the myosin cross-bridges (see the tip region 6 and 5 in No 2 at 3.00 μm sarcomere length). Shorter sarcomeres contact with much more cross-bridges at regions of slower thin filament rotation.

Figure 13 shows the extended diagram of Gordon *et al*. [188]. Under slack-length condition (No 4), with 2.20–2.25 μm sarcomere length, all cross-bridges are in optimal contact with the thin filaments. At shorter sarcomere length of No 5, the situation is hardly changed. Under the condition of No 6 and No 7 the thin filaments overlap and may drill against each other. In this situation the thin filaments may be arranged in a tetragonal pattern as seen in axial direction [215]. Since at 1.65 μm sarcomere length (No 7) the thick filaments collide with the Z-lines, the abrupt velocity decrease of sliding, shown in Figure 12, may occur.

When the sliding motion is considered as a torque-driven drilling rotation of the thin filaments, the quickest rotation should be found at the free ends. Near the Z-band the rotation velocity is near zero. In No 4 of Figure 13 the rotation velocity along a thin filament is expressed by numbers l to 6. The actual sliding velocity should be an average value of all drilling velocities of the thin filament segment within the A-band in contact with cross-bridges. The velocity may be near 4 in No 4. The quicker drilling rotations 5 and 6 of the tip and the slower rotations 3 and 2 should produce friction and frictional heat. This property means that cross-bridges must slip under all conditions, unless the Ca^2+^-activation is incomplete and includes the proximal thin filament regions only. It is shown by the longer sarcomere at 2.7 μm length (No 3), where the sliding velocity starts to increase, that the number of retarding cross-bridges is decreased. At 3.00 μm sarcomere length (No 2), when the sliding velocity strongly increases, cross-bridges are in contact with quickly drilling tips only. The force of the arising resting tension, shown in Figure 12, may work parallel with the increasing sliding force of the tips. Conversely, during shortening of long sarcomeres, the sliding velocity should decrease, e.g. from 3.00 μm sarcomere length to 2.20 μm.

### 3.13. “Quick release”, “Slack-test” and “Force-depression”

**Figure 14:**
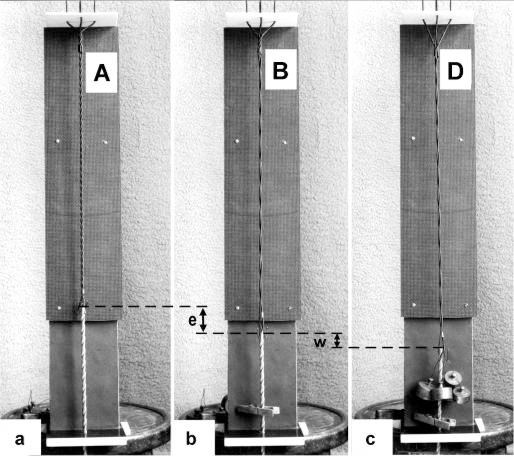
Model that simulates the “contractile” thin filament as a torque-containing component and the series elastic Z-filaments. A double helix of two twisted rubber cords is fitted between the Z-filaments and the borer helix. The model is relaxed in (a), twisted by the additional torque for 16 rotations in (b), and twisted for 16 rotations and loaded by 600 g in (c), that corresponds to the isometric stage D.

To study the behaviour of the series elastic Z-filaments, we used so far the model of Figure 4, where the thin filament is represented by a rigid helix. In order to investigate the two component mechanics, we need a model that contains torque also within the contractile component. It consists of a rubber cord wound about itself and fitted between the Z-filaments and the borer-helix, as shown in Figure 14. A long rubber cord (length about 100 cm, diameter 3 mm) was at first fixed at one end by a vice. Then it was strained under tension by about 60 clockwise rotations of the other end. Now the two halves, each about 50 cm, are hold parallel side by side, and are so slowly relaxed, that they can wind about each other. The relaxed right-handed double helix is fixed with super-clue by one end at the borer and by the other end at the four Z-filaments. The relaxed model is shown in Figure 14a. The A-stage of the Z-filaments is brought about by turning the Z-filaments, as described in chapter 3.2. Then the double helix was again torsionally strained by 16 counter clockwise rotations of the borer. This leads to an increased pitch of the double helix that contains now a torque for about 16 clockwise rotations of the borer (Figure 14b). The pitch decreases again when the double helix rotates, but the rotations are prevented by a clothes-peg in Figure 14b. The torque for about 16 clockwise rotations is now in equilibrium with the torque for about one rotation of the Z-filaments. It untwists the Z-filaments from stage A in Figure 14a to about stage B in Figure 14b. The model of Figure 14b comprises torque but no tensile stress. The isometric state is reached when this model is burden by a load of 600 g (Figure 14c). The tensile stress of the load stretches the Z-filaments to stage D. Hill [39] denoted the stretch of the series elastic component in the isometric state with the letter c. The elongation c after counter-clockwise twisting the relaxed model and the twisting distance W of the Z-filaments (see Figure 5, stage D) results in the elongation shown in Figure 14c. However the soft rubber cord model of Figure 14 is more compliant than the real thin filament. The actin filament together with activated Tm-Tn complex is much stiffer, although tropomyosin can twist and torsionally rotate the model filament. It is not possible to simulate both properties (the high torsional stiffness against stretch and the torsional compliance against the tropomyosin torque) by one and the same model. The high torsional stiffness is better simulated by the model of Figure 4.

It is demonstrated by the rigid wire model of Figure 10 that a strong stretch changes stage A to stage D. The same does the load on the model of Figure 14c. In contrast to the borer helix in Figure 4, which is rotated by shifting the nut-coil, tensile stress works here only. However, tensile stress without rotation can never reach the stages E, F and G, as does the nut-coil model of Figure 4.

Each drilling rotation of a borer-helix produces drilling- or sliding-pressure. The sliding-pressure of the used models was too weak for stretching the Z-filaments e.g. from stage A to D (Figure 10). Stronger stretch was obtained by weights (Figs. 4 and 14). It is possible that the thin filaments of muscle can in relation produce much stronger torque and sliding pressure than the limited torque of the rubber-cord models can do.

It is shown by the model of Figure 14, that the twisting stage of the series elastic Z-filaments depends (1) on the torque of the thin filament, which is in equilibrium with the torque of the Z-filaments. The torque for about one rotation of the Z-filaments corresponds with the torque for many torsional rotations of the thin filament. 16 rotations were used in the model of Figure 14. During the drilling rotation of shortening both torques should slowly decrease. According to Ramsey and Street [61] a sarcomere can contract to l μm length. Therefore the maximum sliding distance of a thin filament is more than l μm (1000 nm). Since 72 nm, the pitch of the thin filament helix, goes about 14 times in l000 nm, at least about 14 rotations of the thin filament must be possible. (2) Moreover, the twisting stage of the Z-filaments depends on the tensile stress that stretches the elastic filaments, as demonstrated by the models of Figs. 10 and 14. A quick stretch or a quick release of muscle must at first change the length of the elastic component [38]. (3) The stages E, F and G are reached only by rotating the thin filaments, as shown by the model of Figure 4, i.e. when a muscle is stretched before or after the activation.

**Figure 15:**
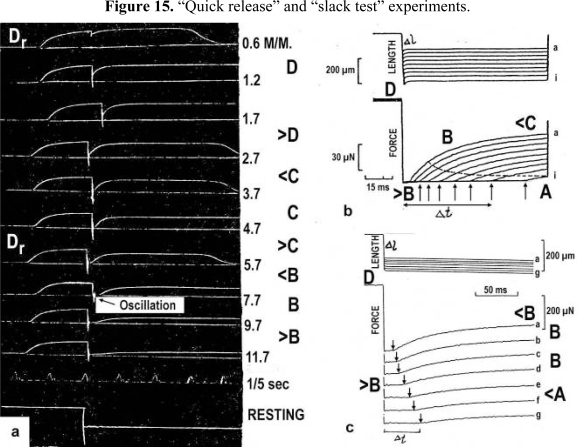
“Quick release” and “slack test” experiments. (a) Quick releases on tension recorded in maximal isometric tetanus of frog’s sartorius muscle. The quick release amplitudes are shown in mm (from Gasser and Hill [216]). The approximative twisting stages of the Z-filaments are indicated, and the position of oscillations is denoted. (b) Nine slack test experiments on a skinned rabbit fibre (fibre length 2000 μm, sarcomere length 2.5 μm). From Galler and Hilber [217]; with kind permission of Springer Science and Business Media. (c) Seven slack test experiments on a single skinned fibre preparation from rat leg muscle (from Galler *et al.* [218]; with permission). The upper panels show fibre length, Δl is the release amplitude. The lower panels show the force. Arrows indicate end of unloaded shortening, Δt is the time to the redevelopment of force. Approximative twisting stages of the Z-filaments are indicated.

First quick release experiments with useful results were performed by Gasser and Hill [216] on frog sartorius (Figure 15a). The tetanus force-curve is interrupted by a quick release of different amplitudes, indicated in mm at the right side. “No matter how small the extent of the release, the tension fell below the isometric value of the shorter length and then rose again, approaching it asymptotically. The fall in tension increases in amount with the extent of shortening allowed, until the latter becomes 10–15 per cent of the total length, at which point the whole of the tension disappears momentarily on release. If larger amounts of shortening than this be allowed, the tension remains at zero a finite time before it begins to rise again, the duration of the phase of zero tension increasing as the amount of shortening increases”. This is the exact description of “quick release” and “slack-test” experiments, but later measurements with improved techniques yielded 1–2 % of muscle length instead of l0–15 % for the disappearance of tension. The lowest 4 force-curves in Figure 15a are already releases with slack test character. Figure 15 b, c shows slack test experiments of skinned mammalian fibres of rabbit (b) and rat (c), [from 217,218]. Striking with all curves of Figure 15 is the increasing “force depression” (force deficit) with increasing release amplitude. The lowest curves rise only quite slowly.

**Figure 16:**
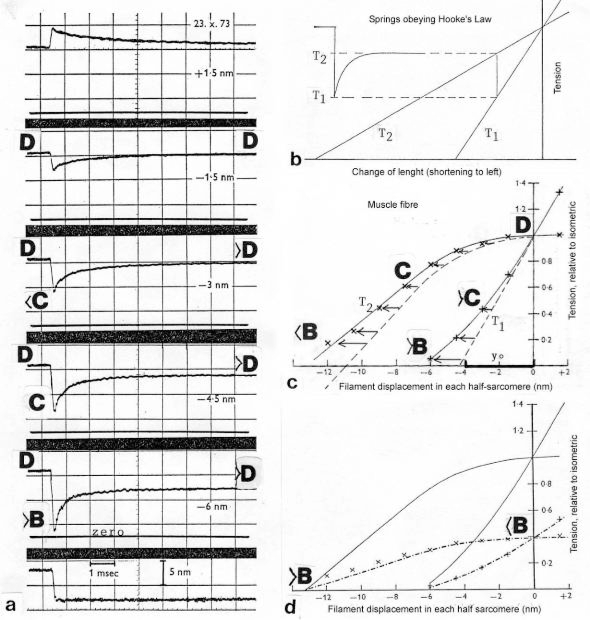
Quick length changes of small amplitudes on isolated frog muscle fibres during tetanic stimulation (all figures from A. Huxley [238]; with permission). (a) Tension curve of a quick stretch 1.5 nm (top panel) and quick releases of 1.5 nm, 3 nm, 4.5 nm and 6 nm. The length change, shown in the bottom panel, is complete in 0.2 ms [219]. (b) Tension transient response in an ideal Voigt element, i.e. spring in series with parallel combination of spring and dashpot. T_1_ extreme tension reached, T_2_ tension approached asymptotically. Both T_1_ and T_2_ give straight lines when plotted against the size of the imposed step change of length. (c) T_1_ and T_2_ curves constructed from a family of records as shown in (a) (sarcomere length 2.3 μm). Approximate changes of the Z-filament stages are added and some possible displacement by drilling is indicated by arrows. (d) The fibre with the T_1_ and T_2_ plots (crosses) shown in (c) stretched to sarcomere length 3.l μm, i.e. the overlap is reduced to approx. 39 %. The tension curves (crosses) scaled down by the factor 0.39 (from Huxley and Simmons [226]).

For the interpretation of the described experiments it is necessary to distinguish between two processes that are both involved during a release: (1) Quick shortening by reducing the stretch of the Hookenian elasticity, as shown by the model of Figure 14, when the load is removed from Figure 14c to b. This shortening comprises maximal about 1 % of muscle length and is mainly (not fully) adequate to the shortening of the series elastic component. (2) Slower shortening by drilling rotation (arrows 3 and 4 in Figure 1) of the thin filaments. The most upper curve of Figure 15a shows a typical release (0.6 mm) of the elastic tension with a nearly complete recovery, and (nearly) no drilling rotation. The redeveloped isometric tension is not reduced. Ford *et al.* [219] have done similar experiments with small releases of 1.5, 3.0, 4.5 and 6 nm per halve sarcomere by using much better time resolution (Figure 16a). They compared the instantaneous tension drop followed by the early tension recovery with the response of a “Voigt element”, i.e. a spring in series with a parallel combination of spring and dashpot (Figure 16b). They plotted the T_1_ values (the extreme fibre tension reached with the length step) and T_2_ values (the tension approached during the early recovery), and found that the T_1_ curve reached the zero line between 6 and 8 nm displacement, and that both curves deviate from a straight line.

**Figure 17:**
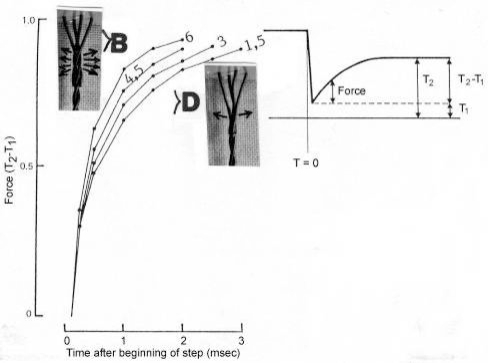
Time course of force recovery following step releases of various amplitudes 6, 4.5, 3 and 1.5 nm. Force is expressed as a fraction of T_2_-T_1_. The step change in length started at time 0 and was complete in 0.2 ms. The speed of the recovery depends on the distance of the length change. It decreases with the release amplitude (Ford *et al.* [221], from Woledge *et al.* [49]; with permission). Z-filament twisting stages are added. The change of the twisting stage e.g. from D to >B during the 6 nm release step, displaces much more Ca^2+^ (arrows) and effects a quicker recovery than the change from D to >D during the 1.5 nm release step.

**Figure 18:**
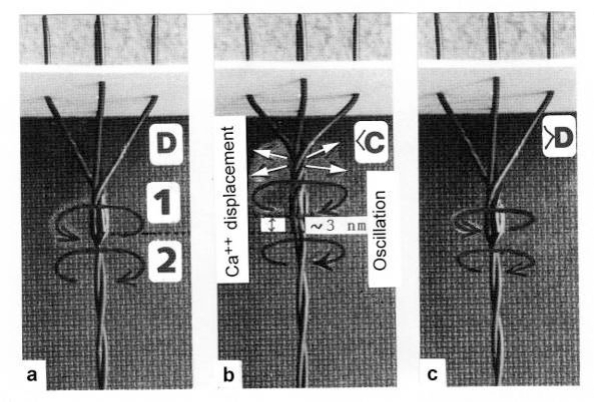
A quick release of small amplitude is simulated by the model of Figure 14. (a) Isometric state, the torques of arrow l and arrow 2 are in equilibrium. (b) A quick release of 3 nm has quickly changed the Z-filament twisting stage, e.g. from D to <C (the twisting stage of the model is here somewhat too large), Ca^2+^ is displaced (arrows) and a damped feedback oscillation can occur. The Ca^2+^ displacement results in a decrease and reversal of the Z-filament torque. Arrow l and arrow 2 work now into the same direction, so that the Z-filaments can again untwist, and the force redevelops. (c) The isometric state with equilibrated torques (arrow l and arrow 2) is again reached. Little torque of the thin filaments is lost. The difference between stage D and >D is not visible.

We can interpret the force curves in detail by using the isometric stage of the model in Figure 14c. It is stretched by the isometric force to stage D (Figure 18a), and the torque of the Z-filaments (arrow 1) is equilibrated with the torque (arrow 2) of the thin filament. A quick release, analogous e.g. to about 3 nm, means a reduction of the elastic stretch and a change of the twisting stage from D to <C (Figure 18b). The winding up of Z-filaments means Ca^2+^-displacement as indicated by arrows and consequently a decrease or reversal of the Z-filament torque. Arrow l points now into the same direction as arrow 2. The torque equilibrium is lost, and after possible damped feedback oscillations, the thin filament torque causes limited rotation (arrow 2) that untwists the Z-filament again to the isometric state with equilibrated torques and a slightly reduced force of stage >D (Figure 18c), because a little thin filament torque is lost. It is again the torque of the prevented contraction (drilling rotation, arrow 3) that produces by rotation (arrow 2) the redeveloped force as also the initial isometric force. The rise of the redeveloped force must be connected in any case with untwisting the Z-filaments, e.g. from stage <C to >D. It was shown by Jewell and Wilkie [220] that the redeveloped tension after a quick release arises quicker than the initial rise of tension. Ford *et al*. [221] applied releases from 1.5 nm to 6 nm, as shown in Figure 17 and found the recovery was more rapid from large releases than from small ones (Figure 17). A large release displaces more Ca^2+^ than a small one, as indicated by the small model-photographs in Figure 17. Thus the untwisting motion of the Z-filaments takes place quicker after large releases.

**Figure 19:**
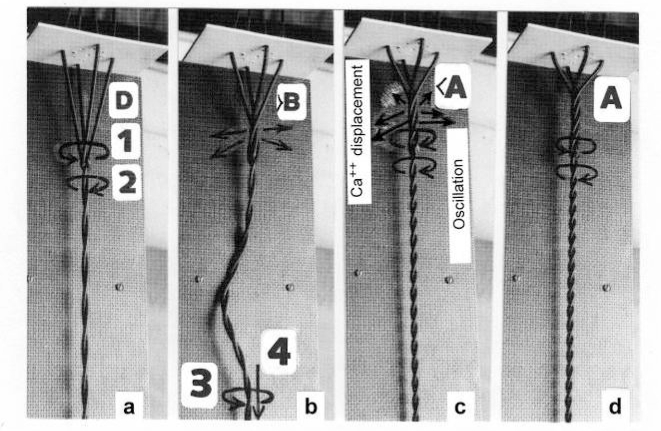
Quick releases of a high amplitude simulated by the model of Figure 14. (a) Isometric state, the torques 1 and 2 are equilibrated. (b) During the release the Z-filament twisting stage increased transiently e.g. to >B, when the zero point of the Hookenian elasticity is passed over and the thin filament becomes slack. But the thin filament torque has started rotation (arrow 3), and drills the thin filament into the myosin cross-bridges (arrow 4), as long as shortening is stopped. (c) When shortening is stopped, e.g. at the Z-filament twisting stage <A, Ca^2+^ was displaced already in stage >B, resulting in feedback oscillations and reversal or reduction of torque (arrow 1), so that the increasing thin filament torque (arrow 2) can again untwist the Z-filaments and produce force redevelopment. (d) A second release, e.g. in a slack test, can further relax to stage A, where a torque equilibrium may be not more possible (comp. Figure 15 b, c). The strong force-deficit or torque-loss is here shown by the decreased pitch of the model thin filament, that does not so occur in real thin filaments.

Now to the drilling rotation of the thin filament during a quick release. Figure 19a shows again the stretched isometric stage D with equilibrated torques. In the moment of release (Figure 19b) the Z-filaments twist e.g. to stage >B. Ca^2+^ is displaced (arrows), and the thin filament has lost its tensile stress as it is excessively shown by the slack stage of Figure 19b. But the torque in the thin filament is effective, and rotation (arrow 3) and drilling (arrow 4) starts instantly. Little drilling rotation should occur (arrows in Figure 16c) with small releases that may increase the nonlinearity of curves T_1_ and T_2_. During quick releases and slack tests with large amplitudes (Figure 19) the thin filaments can perform some rotations and may shorten the muscle fibres with V_max_ until they are stopped, e.g. in stage <A (Figure 19c). A second small release can then lead nearly to stage A where the torques can attain their equilibrium (Figure 19d). The pitch decrease of the double helix in Figure 19c, d indicates the torque-loss. Ca^2+^ displacement should occur after shortening and can produce some oscillations indicated in Figures. 18b and 19b, c.

The slack tests on skinned fibres of Figure 15b, c leads at first to a quick relaxation of the tensile stress, indicated by the vertical slack length Δl. This can lead e.g. from stage D (Figure 19a) to >B (Figure 19b). Together starts the drilling rotation (arrows 3 and 4) of unloaded shortening, indicated by the horizontal slack time Δt. Arrows show the end of the unloaded shortening in Figure 15b, c and the start of the redeveloped force. During the elastic release the torque reservoir of the thin filaments remains largely intact, but the drilling rotations reduce it vigorously. The redeveloped force attains its maximum in the quick releases of Figure 15a, but not in the slack tests of Figure 15b, c. The diminished torque of the thin filaments means also diminished torque of the Z-filaments. Thus the resistance of the Z-filaments against untwisting may strongly decrease. The force of the lowest curves of Figure 15b, c may hardly reach a quarter of the original force. The fall of the force with increasing slack time Δt is indicated by the dashed line in Figure 15b. The slope of the redeveloped slack test curves is steep at first, but decreases with time, indicating further torque-loss. The non parallel curves at the start means shorter distances at the x-axis between shorter slack times (Figure 15b). Therefore slack time Δt plotted against slack amplitude Δl results in two- or three-phasic curves when shorter slack times are included [217].

**Figure 20:**
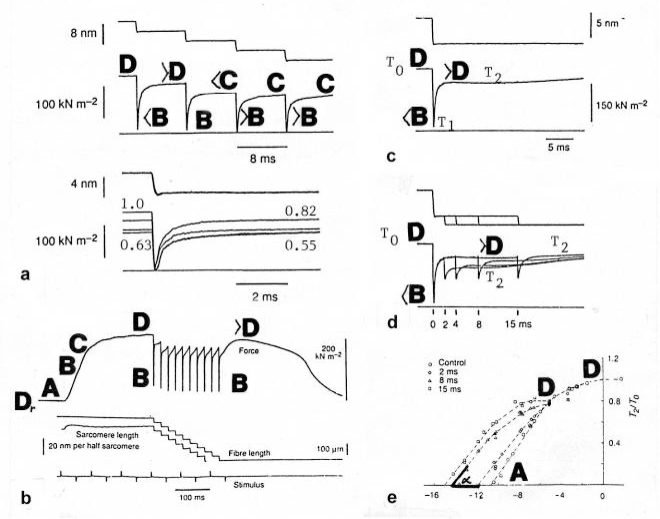
Quick release experiments and force-depression. The approximative Z-filament twisting stages are indicated. (a) A four-step release (Lombardi *et al.* [222], with permission). A model simulation is tried in Figure 21. Only the first two steps distinctly show force depression. (b) Ten quick shortening steps of 0.12 ms (Irving *et al.* [223], with permission). No distinct force-depression. (c) One step release with distinct force-depression [222]. (d) and (e) Five-step release [222]. The force-depression disappears with the steps. For further details see text.

**Figure 21:**
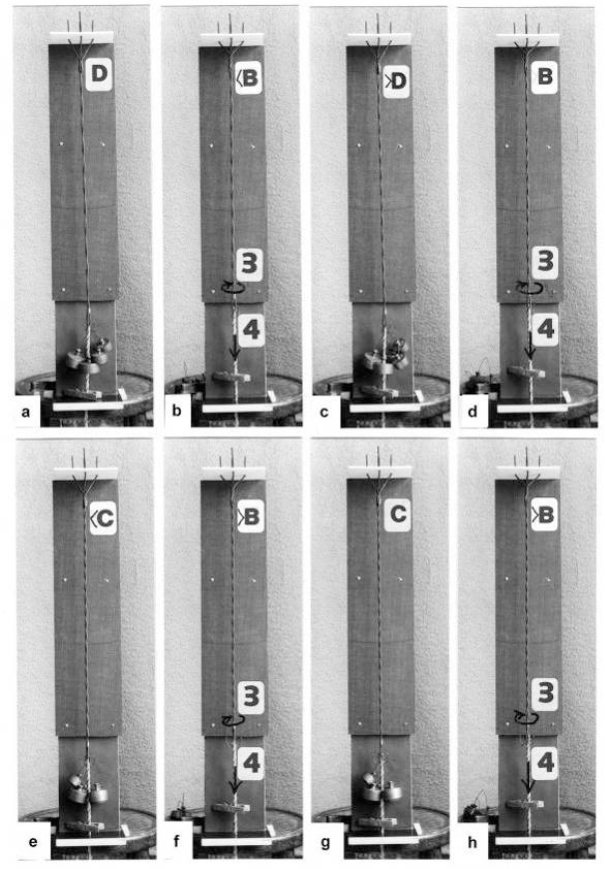
Model-simulation of the four-step release in Figure 20a. The approximative Z-filament twisting stages are indicated. For more details see text.

A four-step release experiment on intact frog fibres was performed by Lombardi *et al*. [222], as shown in Figure 20a. It is coarsely simulated by the double-helix model of Figure 14. Figure 21a may accord with stage D at the plateau of an isometric tetanus. The first quick release of 4.15 nm per half sarcomere (Figure 21a → b), performed by removement of the weights, leads to a transient twisting stage <B and to elastic shortening of the filaments. In the moment of release the relieved filament starts clockwise rotation (arrow 3) and drilling into the A-band (arrow 4). The force redevelops as described in Figure 21b, c, leading to the twisting stage >D (Figure 21c), where the redeveloped force is simulated again by addition of the load. The same procedure is three times repeated. The letters denoting the twisting stages of Figure 21 are approximately indicated in Figure 20a. There is a torque-loss after each release step by the thin filament rotation during the redevelopment of the force.

The amount of the rotations was exaggerated in order to make it more distinct at the model: 2 rotations between a and b, and between c and d, 1 rotation between e and f, and between g and h. Together with the torque-loss the Z-filament twisting stage increased a little, not visible in Figure 21h. The double helix pitch however, conforming to the actin helix pitch, is distinctly decreased by the torque-loss in Figure 21h, as compared with Figure 21b. 6 Rotations torque-loss performed at the model is certainly too much for about 17 nm total release per half sarcomere. The actual torque-loss in the experiment of Figure 20a may be less, perhaps 2 rotations. It is here demonstrated that shortening under isometric conditions depend on “inner” processes (arrow 2), that are different from isotonic shortening, where the thin filament pitch 72 nm times rotation number, may be valid for the amount of shortening. However, when cross-bridge slippage occurs, the number of rotations can be higher than the number of the pitch-displacements.

A second experiment on intact frog fibres of 10 quick shortening steps, each of duration of 0.12 ms and an amplitude of 6 nm per half sarcomere, at 20 ms intervals [223], is shown in Figure 20b. In spite of about 60 nm total release the force depression is small (D → D). This may depend on the prematured interruption of the force redevelopment that stops the drilling rotation of the thin filaments early after each shortening step. A second important factor is the extra Ca^2+^ that is set free by each quick release (see chapter 3.7) and may compensate the torque-loss by increased Ca^2+^ binding in the Huxley-Simmons phase 4.

A further quick release experiment on intact frog fibres was done by Lombardi [222], and is shown in Figure 20c, d, e. With a first conditional step release of about 5 nm the tetanic tension drops close to zero (<B, a similar release step is simulated at the model of Figure 21b). A quick recovery is completed within about 2ms, leading to a force depression of about 20 % (stage >D in Figure 20c). During the next 13 ms tension changes less than 2 %. Now test release steps of 2 nm were applied 2, 4, 8 and 15 ms after the conditional step (Figure 20d). Its force depression decreases with time and is completely lacking after 15 ms. This has been interpreted as rapid regeneration of the power stroke according to the swinging cross-bridge theory. However, it was shown that quick releases of high amplitude always produce transient extra Ca^2+^ (displaced by winding up of the Z-filaments) with peaks 50ms or more after the quick release. Thus, the force depression after the later test steps in Figure 20d may be compensated by an increased Ca^2+^ binding when the Ca^2+^-concentration is elevated (see chapter 3.14). Hill described in some papers that “in an isometric twitch, 15–20 % of the whole energy liberated is due to a sort of positive feed-back during the decline of the active state. The tension existing at any moment during the falling phase works somehow to prolong activity”. “…the arrangement reminds one of a system in which a displacement from rest tends to prolong itself by positive feed-back” [68, p. 81, p. 140–41, p. 362] and [224].

Lombardi *et al*. [222] obtained T_2_ curves (Figure 20e) of the shortening steps described in Figure 20d. “An interesting feature” of the T_1_ and T_2_ curves is, “that they approach the base line at a definite angle and not tangentially or asymptotically” [quot. 238]), as shown by the angle α indicated in Figure 20e. This was interpreted by Huxley in accordance with his contraction theory, that the cross-bridges are able to exert negative tension. Blangé *et al*. [225] claimed that the curve can approach the base line tangentially, but this is not the case, as shown by Figure 20e. A better interpretation is that the T1 and T2 plots depend not only on the elastic release but also on some drilling as indicated approximately by the arrows in Figure 16c. The torque responsible for the drilling rotation is not zero at the zero-line of the elastic relaxation.

### 3.14. The Huxley-Simmons phases during quick length changes

**Figure 22:**
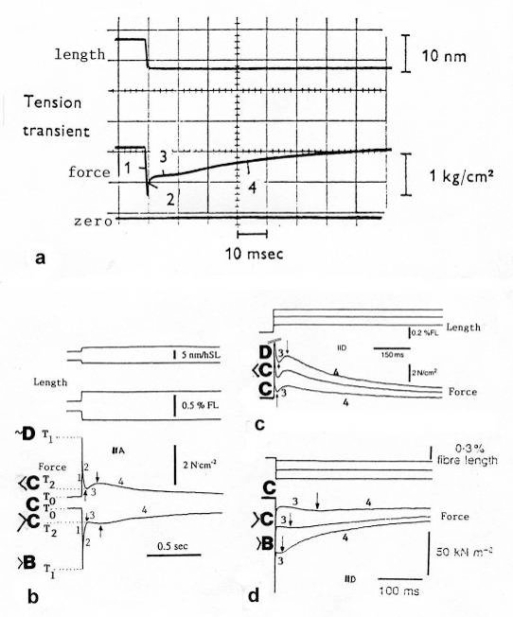
The four Huxley-Simmons phases after quick stretch and release. (a) Tension transient of an isolated intact frog fibre after a small quick release (from Huxley [238]). (b) Quick stretch and quick release of a chemically skinned human muscle fibre (from Hilber and Galler [227]). (c) Quick stretch experiments on a skinned rat leg fibre (from Galler *et al.* [228]). (d) Quick release experiments on a skinned rat leg fibre (from Galler *et al.* [229]; with permission, b and c with kind permission of Springer Science and Business Media). Phase l: Elastic extension (during quick stretch) or relaxation (during quick release). Phase 2: Delayed passive thin filament drilling. Stretch: counter clockwise rotation that increases the original torque. Release: clockwise rotation that decreases the original torque. Phase 3: Delayed force increase after stretch by additional Ca^2+^ binding on the Z-filaments during unwinding in the region from stage A to D. Force decrease after release by Ca^2+^-displacement from the Z-filaments wound up in the region from stage D to A. Phase 4: Slow force decrease after stretch, presumably by tropomyosin shift dependent Ca^2+^-displacement from the thin filaments. Slow force increase after release, presumably by Ca^2+^-binding on the thin filaments. Note the amplitude-dependent steeper inclination in (c) and (d). Some of the extra Ca^2+^ displaced during release in phase 3, may be bound by the thin filaments, prolonging the active state.

The four phases or “tension transients” described by A. Huxley [192, 238] are shown in Figure 22a after release of a tetanized isolated frog muscle fibre. Figure 22b recorded stretch and release of a skinned human fibre [227], Figure 22c and d stretch and release of a skinned rat fibre [228, 229].

The elastic phase 1 of release produces loss of the Hookenian tension from T_0_ to T_1_ (Figure 22b) and is simulated by the transition from a to b at the models of Figure 18 and 19. The Z-filament twisting stage may increase e.g. from D to >B in Figure 19b. In intact muscle this can occur in the first millisecond. Ca^2+^ displacement lasts longer. The thin filament becomes slack by the quick relaxation (Figure 19b), but its torque starts phase 2 by friction-retarded clockwise drilling rotation (arrows 3 and 4 in Figure 19b), that makes it taut again. Torque is reduced by this clockwise rotation, and the twisting stage of the Z-filaments increases e.g. from >B to <A (Figure 19c, “force deficit after release”). Therefore the rising tension can increase only to T_2_ (Figure 22b), where a tension decrease of phase 3 occurs owing to Ca^2+^ displacement. But since Ca^2+^ displacement means a torque decrease of the Z-filaments and abolition or reversal of arrow l (Figs. 18b and 19c), the restricted rotation (arrow 2) of the thin filament torque becomes effective and can now easily untwist the Z-filaments, and completes the redevelopment of force (phase 4 in Figure 22b) by reaching nearly the initial tension stage. But some torque is lost. It is striking that the long lasting phase 4 increases strongly after larger release amplitudes (Figure 22d), whereby phase 3 becomes insignificant by phase 4 super-position. During reduced muscle tension the two tropomyosin coiled-coils may be less strongly pressed on the actin filament. Thus more Ca^2+^, displaced as extra Ca^2+^ from the Z-filaments after the release, may be bound on it and may increase the thin filament torque. This slowing of relaxation or “positive feedback” [224, 230] could be a mechanism for emergency to use all the free Ca^2+^ ions for activation when the force is already low.

During the elastic stretch of a fibre in phase l the Z-filaments are quickly changed within one millisecond, e.g. from stage C to ~D (or the stage D becomes more stretched). The tension reaches the extreme stage T_1_ (Figure 22b). The thin filaments are thereby passively drawn out off the myosin cross-bridges. But this motion is a friction-retarded counter clockwise drilling rotation (phase 2) of the thin filaments that decreases the extreme tension T_1_ again to T_2_. The tension level of T_2_ is higher than T_0_ because the counter clockwise rotation has increased the torque (“stretch induced force enhancement”). Also the twisting stage should increase again, e.g. from D to <C. The initial change of the Z-filaments from stage C to ~D has caused increased Ca^2+^-binding that is delayed, but still increases the torque of the Z-filaments (phase 3). That increased tension of phase 3 after stretch corresponds to the negative curve of extra Ca^2+^. Moreover, the increased force during stretch may press the tropomyosin coiled-coils stronger to the actin filament surface. This could displace Ca^2+^ ions and may cause the long lasting phase 4, that decreases the force (Figure 22c), especially when the stretch amplitude was large.

If a muscle is slowly stretched under counter-clockwise rotation of the actin filaments, the Z-filaments can reach stage E, F or G. In this region stretch from stage D to G produces strong passive torque by counter clockwise thin filament rotation, but less active torque, because extra Ca^2+^ is displaced. Thus Lombardi and Piazzesi [231] found reduction or disappearance of phase 3 during steady lengthening. Release from G to D means increased Ca^2+^ binding that should increase the active torque. These torque effects are contrary as indicated in Figure 5. The “region of addition” is here between A and D, the “region of compensation” between D and G. The irregular behaviour of muscle between D and G is also expressed by the divergent stiffness in that region (see Figure 24c).

The Huxley-Simmons phases are effected by MgATP concentration [232,233]. The speed of the phases and the ATPase activity increases with the MgATP-concentration of the bathing solution. But the rate constants of phase 2 showed a dependence on the amplitude of the length change, and did not depend on Ca^2+^ and MgATP-concentration [234]. This confirms that the filament rotation during phase 2 is a passive effect after stretch or release.

### 3.15. Shortening after sudden load reduction

**Figure 23:**
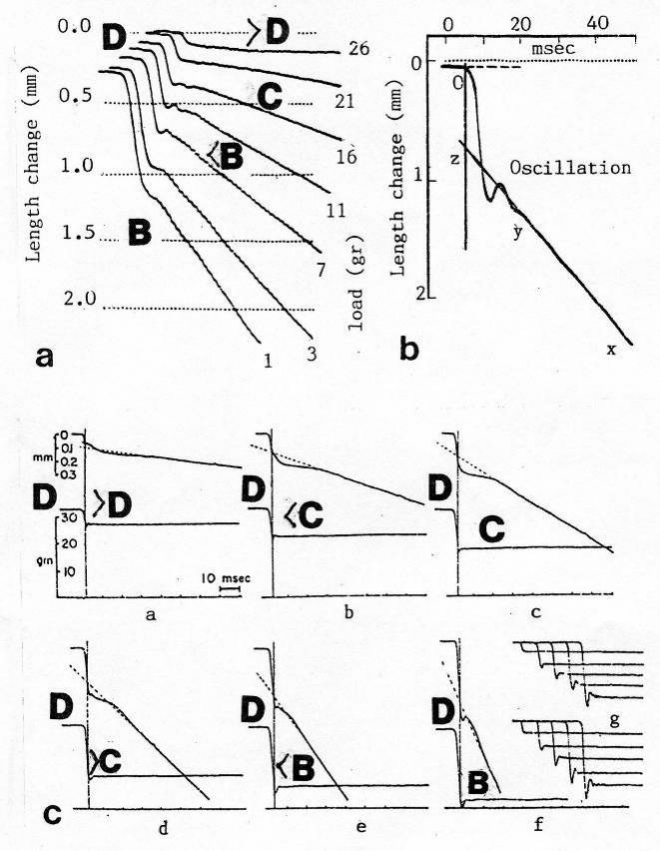
“Quick displacement” and isotonic steady state shortening after sudden load (force) decrease of a tetanized frog muscle. The approximative Z-filament stages are indicated. (a) The difference between quick displacement of the “series elastic component” and the steady state shortening of the “contractile component” is striking. The length change (left, in mm) and the remaining after-load (g) is indicated near each curve. (b) Damped oscillations superimpose the beginning of steady state shortening ((a) and (b), improved from Jewell and Wilkie [220]). (c) Similar experiments with own (lower) curves for the remaining afterload. At right in the lower panel, the tension curves of a spring which was fixed instead of the muscle (from Podolsky [236], with permission).

The load of a tetanized muscle under isometric conditions is suddenly reduced and the length-change recorded. Experiments of this kind were at first done by Wilkie [235], Jewell and Wilkie [220] and Podolsky [236]. Figure 23a [from 220] shows at first sight the division of the shortening curves in two parts that mirror the two components of muscle: (1) The first “quick displacement” as the elastic shortening of the stretched series elastic component, and (2) the straight curve of the isotonic steady state shortening of the contractile component. The load (g) is indicated near the right end of each curve. The beginning of the steady state shortening is superposed by damped oscillations (Figure 23b, from [220]), which were later also described by various authors [237–239]. The slope of the straight steady state curves depends on the load. Podolsky’s experiments [236] (Figure 23c) show the length change as the upper curves separated from the lower force (load) curves. Civan and Podolsky [240] found with improved techniques that the quick displacement lasts 1–2 ms.

The interpretation is again possible with the help of the model in Figure 14. The first step is the same as with a quick release (Figure 18 a to b). The filaments shorten elastically and the Z-filaments wind up, e.g. from stage D to <C (Figure 18b). The amount of the quick displacement depends on the amount of load reduction. In the moment of the release the thin filaments start clockwise drilling rotation. But from stage <C, the drilling rotation must lift the remaining load. The velocity is quicker and the slope steeper when the load is reduced. The Ca^2+^ displacement, shown in Figure 18b can produce damped oscillations that superpose the beginning of the steady state shortening. The first deflexion may correspond to the falling force of phase 3. The extrapolation done in Figure 23b and c by lengthening the line x - y to the zero line in z, may not be correct, because the thin filament should start its drilling rotation in the moment of the release.

When the load is suddenly reduced on skinned fibres the reduced force is constant as in Figure 23c, but the isotonic shortening velocity slows down during shortening as indicated by upward concavity of the curves (e.g. Figure 1C in [241]). Such slowing down of the shortening velocity is also possible on intact fibres [188]. It seems to be the result of a low Ca^2+^ activation. The same slowing down of the isotonic velocity was observed by Brenner after a slack test on skinned fibres [242]. The slowing down is sometimes quicker at the beginning of the shortening curves. This can be explained by the quicker rotation of the distal parts of the thin filaments that are at first contacting the thick filaments (see chapter 3.12 and Figure 13, No 2). We interpret the progressive force depression during isotonic shortening of skinned fibres by a continuous torque-loss of the drilling thin filaments. When this slowing down of the velocity is prevented by imposing isovelocity shortening after a quick release, the force slows down [242]. This strongly speaks in favour of a continuous torque-loss during shortening.

### 3.16. Force depression, force enhancement and cross-bridge slipping during a length change

Release of an activated muscle causes depression (deficit) of the redeveloped force [243 for review], because the thin filament torque has been diminished by the clockwise drilling rotations during shortening. Stretch of muscle causes residual force enhancement (“stretch activation”) as reviewed by Herzog *et al*. [244], because the thin filament torque has been increased by the counterclockwise drilling rotation of the thin filaments, when they are drawn out off the cross-bridges (see Chapter 3.1). Residual force enhancement after stretch may depend also on nonuniformities of sarcomere lengths [244], but it is also found with uniform length of the sarcomeres [245]. The effect of stretch is very complex when the elastic extension is considered. Figure 10 shows that a sudden stretch can transiently change the Z-filament twisting stage to more Ca-binding and more torque, which may participate in the transient tension maximum before the tension falls to the residual enhancement.

**Figure 24:**
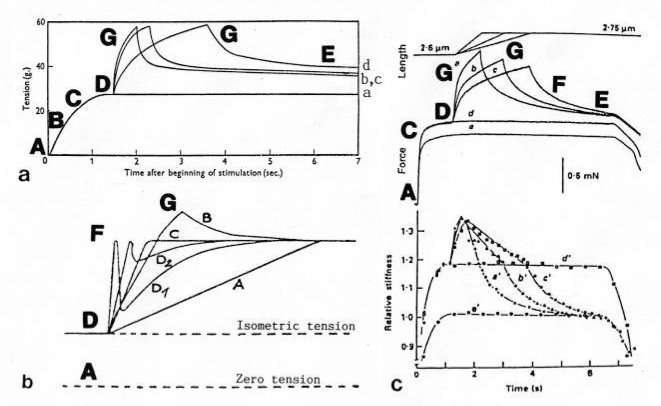
Stretch activation in the high-tension region between Z-filament stage D and G. The approximative Z-twisting stages are indicated. (a) The force enhancement depends on the stretch velocity. “The quicker the stretch the less the final excess above the isometric tension” (toad sartorius at 0°, from Abbott and Aubert [246], with permission). (b) Dependence of the tension curve after stretch on stretch velocity. A: very slow (“reversible” stretch), D_1_: very rapid, D_2_: rapid, B: fairly rapid, C: intermediate between B and D (frog sartorius, from Gasser and Hill [216]). (c) Tension (upper curves) and stiffness (lower curves) show irregular behavior (frog’s skeletal muscle, from Sugi and Tsuchiya [250], with permission). For more detail see text.

The amount of force enhancement depends on the amplitude, when the thin filaments are drawn out off the cross-bridges (see Chapter 3.1). But the amount of force depression or force enhancement depends also on the velocity of release or stretch. Classical experiments were performed by Abbott and Aubert [246]. The slowest stretch shows most force enhancement (curve d in Figure 24a). “The quicker the stretch the less the final excess above isometric tension” [246, p. 81]. A quick stretch stresses the cross-bridges more strongly and can produce more slippage [247]. During stretch cross-bridge slipping means force depression by torque-loss. A quick stretch shows more slippage and results in more torque-loss than a slow one. But a quicker release reduces the torque-loss as compared with a slow one. The quick unloaded shortening is the natural velocity. Cross-bridge slipping and torque-loss should increase with the velocity difference between unloaded shortening and the velocity of release. Thus a slow release reduces the tension more than a quick release [246].

As described in Chapter 3.12 (Figure 13), slipping of cross-bridges may be present under all shortening conditions, because the rotation velocity of the thin filaments is highest at the tips. This may bring about cross-bridge slipping also in the isometric steady state.

In the stretch experiments of Figure 24 the Z-filament twisting stages are already in the high-tension region between stage D and G. In this region the muscle shows irregular behaviour that is described more in detail in chapter 3.17.

### 3.17. The Z-filaments in the region of high tension between stage D and G

**Figure 25:**
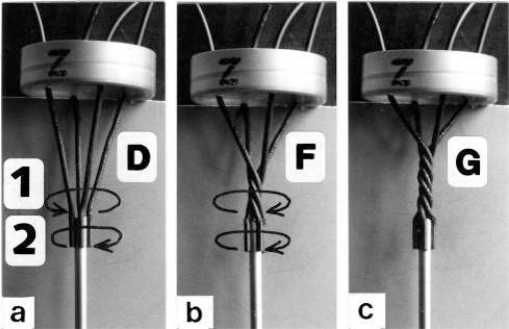
The behavior of the Z-filaments in the high-tension region between stage D and G is different as compared with the tension region between stage A and D. (a) The Z-filament torque (arrow 1) and thin filament torque (arrow 2) is balanced in the isometric stage D. (b) Quick stretch in stage D causes anticlockwise winding up of the Z-filaments (stage F) and Ca^2+^-displacement, that decreases and reverses the Z-filament torque (arrow 1). This results in further anticlockwise winding of the Z-filaments and further Ca^2+^-displacement, leading to an extreme stage G. (c) Stage G, the “limited extent of pull out”, could mean torsional stress for the Z-filaments, perhaps with not provided side-chain discharge and torque reduction

When a load equal to maximum isometric tension (P_0_) is suddenly applied during shortening under low load (0.1 P_0_), the fibre elongates very fast (at several times V_max_), but only for a limited distance, about 100 nm per half sarcomere [51, 52]. This pull out can reach the Z-filament twisting stage G, the “limited extent of pull out”, as shown in Figure 5. In this region the muscle reacts irregularly as compared with the region of normal tension between stage A and D. For example, a stretch from A to B, C, or D means untwisting of the Z-filaments, more Ca^2+^ binding, and increase of the force. A stretch from D to E, F or G (Figure 24) means increased mechanical torque but decreased Ca^2+^ binding, because Ca^2+^ is displaced. A quick stretch from D to F (Figure 25 a to b) leads to Ca^2+^ displacement, decrease or reversal of arrow 1, that should lead to further counter clockwise twisting of the Z-filaments to stage G in Figure 25c, and again to further Ca^2+^ displacement. As a reaction of such a quick stretch torque-loss and untwisting of the Z-filaments may take place with a strong fall of force.

It was shown [216] that a quick stretch applied to a muscle in the isometric state causes at first strong increase and then a quick fall of force, followed by redevelopment (curve D1 in Figure 24b). Here, cross-bridge slipping could be involved. A fairly quick stretch reduces the fall of force (curve D2). The force fall is absent after a slow stretch (curve C), fairly slow stretch (curve B) and very slow stretch (curve A). The curves of fairly slow stretches (curve B) are similar in all experiments of Figure 24. Gasser and Hill [216, p. 435] mentioned: “We are at a loss to explain the fall in tension found by Seemann after stretching”. Seemann [248] stretched the muscle strongly before activation. During the twitch he stretched again suddenly and strongly, and found a considerable increase with following fall of tension to nearly zero, without redevelopment. Perhaps, this was a full torque-loss (discharge of the Z-filaments?) after an extreme strong and quick stretch to stage G.

A second example of irregularity can be found by a release. A normal release, from stage D to C, B or A always decreases the force. However a pre-stretched muscle activated e.g. in stage E or F, and then released to D, can produce increased force [249], presumably since the Ca^2+^ bound in stage D generates higher force than the stretch to stage E or F.

A third example was described by measuring force together with stiffness [250]. Force and stiffness go always parallel in the region between stage A and D [251]. Sugi and Tsuchiya [250] applied a slow stretch on a tetanized fibre (Figure 24c). Stiffness (the curves of the lowest panel) arises at first abruptly, than follows a gradual decrease although stretch is still going on. The final stiffness level is below the initial stiffness, while the isometric force after the stretch is appreciable higher than the expected force at the stretched fibre length. Possibly the first stretch brought the Z-filaments from stage D to E or F (Figure 25b), followed by Ca^2+^ displacement, torque reversal and passage to stage G (Figure 25c) with further Ca^2+^ displacement. The first strong fall of stiffness, still during the stretch, was presumably the result of cross-bridge slipping, followed by a slower decrease, when unwinding of the Z-filaments and again Ca^2+^ binding occurs. The approximate positions of the Z-filament stages are indicated in Figure 24.
